# Hydration Structures on γ-Alumina Surfaces With and Without Electrolytes Probed by Atomistic Molecular Dynamics Simulations

**DOI:** 10.1021/acs.jpcb.2c06491

**Published:** 2022-11-02

**Authors:** Olivera Drecun, Alberto Striolo, Cecilia Bernardini, Misbah Sarwar

**Affiliations:** † Department of Chemical Engineering, University College London, London WC1E 7JE, United Kingdom; ‡ School of Chemical, Biological and Materials Engineering, University of Oklahoma, Norman, Oklahoma 73019, United States; § Johnson Matthey Technology Centre, Sonning Common, Reading RG4 9NH, United Kingdom

## Abstract

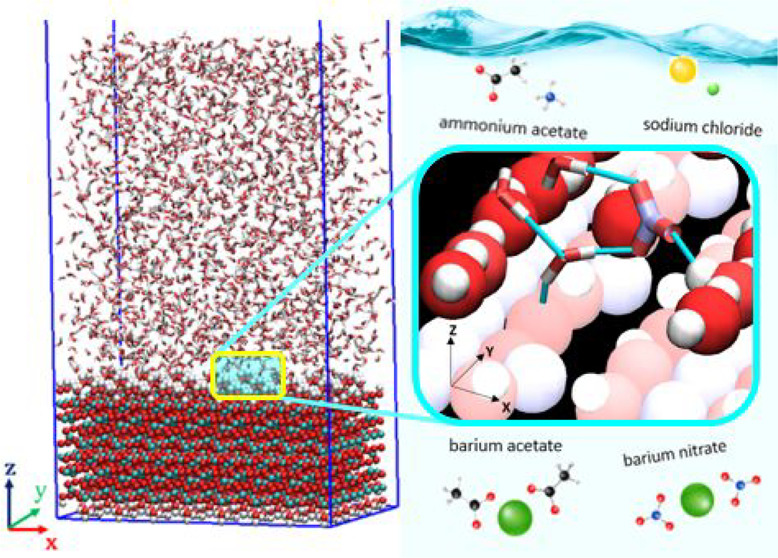

A wide range of systems, both engineered and natural, feature aqueous electrolyte solutions at interfaces. In this study, the structure and dynamics of water at the two prevalent crystallographic terminations of gamma-alumina, [110] and [100], and the influence of salts—sodium chloride, ammonium acetate, barium acetate, and barium nitrate on such properties—were investigated using equilibrium molecular dynamics simulations. The resulting interfacial phenomena were quantified from simulation trajectories via atomic density profiles, angle probability distributions, residence times, 2-D density distributions within the hydration layers, and hydrogen bond density profiles. Analysis and interpretation of the results are supported by simulation snapshots. Taken together, our results show stronger interaction and closer association of water with the [110] surface, compared to [100], while ion-induced disruption of interfacial water structure was more prevalent at the [100] surface. For the latter, a stronger association of cations is observed, namely sodium and ammonium, and ion adsorption appears determined by their size. The differences in surface–water interactions between the two terminations are linked to their respective surface features and distributions of surface groups, with atomistic-scale roughness of the [110] surface promoting closer association of interfacial water. The results highlight the fundamental role of surface characteristics in determining surface–water interactions, and the resulting effects on ion–surface and ion–water interactions. Since the two terminations of gamma-alumina considered represent interfaces of significance to numerous industrial applications, the results provide insights relevant for catalyst preparation and adsorption-based water treatment, among other applications.

## 1 Introduction

Solid–liquid interfacial phenomena are relevant across the fundamental sciences, in influential, if not key areas of understanding from environmental processes^1^ to numerous industrial applications.^2^ Of the latter, one example is the formulation of coatings for heterogeneous catalysis.^3−6^ In the context of heterogeneous catalysis, gamma-alumina is used extensively as a catalyst support material due to a favorable combination of morphological, thermal, and other properties.^7,8^

The present work focuses on (1) the structural arrangements and dynamics of pure liquid water at interfaces of gamma (γ)-alumina, and (2) the effect of salts on the properties of interfacial water.

Experimental studies have investigated γ-alumina/water interfaces in a range of contexts, including, but not limited to: stability of γ-alumina and Ni/Pt γ-alumina supported catalysts under conditions relevant for biomass reforming^9,10^ and at ambient pressure,^11^ sorption of trace environmental contaminants,^12,13^ and radioactive waste containment.^14^ Such studies frequently necessitate the use of in situ/operando analysis techniques.^15−17^ Theoretical and computational approaches can provide synergistic insights at atomistic resolution, assuming that the models implemented are reliable. Of the computational approaches available, studies of γ-alumina surfaces to-date are dominated by density functional theory (DFT)^18−25^ and ab initio molecular dynamics (AIMD)^26−29^ for nonaqueous and aqueous systems; studies of the latter are fewer, and mostly the domain of AIMD. While the level of resolution accessible to DFT and AIMD is exquisitely detailed, the system sizes and time scales attainable using these methods remains limited due to the high computational demands.

To access larger system sizes and longer simulation times (up to 100s of ns), classical molecular dynamics (MD) simulations have been utilized to study interfacial aqueous systems on a wide spectrum of substrates, including oxides,^30−36^ clays,^37−40^ and carbonates,^41−44^ among others. MD simulations discovered fundamental properties, such as the effect of surface polarity on wettability,^45^ the effect of surface patterning on the hydration structure,^46^ and how the dynamics of interfacial water depend on surface features.^47^ Within this landscape, however, studies of γ-alumina utilizing classical MD remain scarce. MD simulations reported to-date for γ-alumina have explored surface structure and rearrangements,^48,49^ glycerol diffusion in nanopores,^50^ structure and dynamics of aqueous isopropanol at the γ-alumina interface^51^ and, recently, thermophysical properties of aqueous nanoparticle suspensions, at low volume-fraction.^52^

The scarcity of classical MD investigations on this system can be attributed to the “defective” structure of γ-alumina, and the resulting debate over which structural model is most representative.^53,54^

From the current state of knowledge, we construct hydroxylated [110] and [100] facets of γ-alumina, based on crystallographic information from the literature. The hydration structure and dynamics of various aqueous phases at both surfaces is then investigated via atomistic MD simulations. Starting with pure water at the γ-alumina surfaces, and establishing effects of the surface features, we then investigate effects on the interfacial hydration layers due to various salts present in the aqueous phase. We consider aqueous solutions of sodium chloride, ammonium acetate, barium acetate (1 molar), and barium nitrate (0.3 molar), building on our prior results for bulk aqueous salt solutions.^55^ These salt systems are relevant for catalyst preparation, as explained elsewhere.^55^ The salt concentrations chosen for the present study were large enough to allow us to probe salt-induced effects at interfaces, yet within the water solubility limit of the various salts. Because oxides—such as γ-alumina—are relatively inert, we assume that electrostatic interactions between the surface and the liquid phase, via surface OH groups, are the predominant mechanism affecting molecular structure and dynamics within the hydration layers. This assumption is reinforced by studies previously conducted on related oxides and the resulting agreement with experimental observations.^56^

Combining several analysis methods, we aim to obtain insights into the interfacial hydration structure, ion-specific effects, and the relation to the surface morphologies of two prevalent crystallographic surfaces of γ-alumina. The remainder of the manuscript is organized as follows; simulation methods and algorithms are described in Section 2, results are presented and discussed in Section 3, followed by a summary of our conclusions, in Section 4. We provide extensive additional results as Supporting Information (SI).

## 2 Computational Details

### 2.1 Methods

All simulations were performed using the freely available software LAMMPS^57^ (version 16 Mar 2018). The velocity Verlet algorithm^58^ was implemented to integrate the equations of motion, with a 1 fs time step. Simulations were conducted with periodic boundary conditions in the canonical ensemble: constant number of particles (*N*), volume (*V*) and temperature (*T*), maintained by the Nosé–Hoover thermostat^59,60^ (100 fs damping parameter). Simulations were conducted at 293.15 K, representative of ambient conditions. As the net charge of all our simulated systems is zero, long-range electrostatic interactions were treated with the particle–particle–particle–mesh (pppm) solver.^61^ The systems were equilibrated for 30 ns, followed by a 4 ns production run. For the analyses presented herein, the production run trajectories were sampled every 400 fs. On the basis of prior experience and observation of aqueous interfacial systems modeling, 30 ns is sufficient for equilibration.^36,40,62−64^ However, it is possible that both simple and complex ions slow the dynamics of interfacial water. To ensure that the equilibration time was sufficient, we monitored the potential energy of the systems, which plateaued within 1 ns of the equilibration simulations, confirmed that water density profiles perpendicular to the surfaces considered did not vary substantially when sampled at 10, 20, and 30 ns of simulation time, and ensured that the ions adsorbed at the interface could relocate to different preferential adsorption sites, and desorb to the bulk water, during the time of our simulations.

### 2.2 γ-Alumina: Crystallographic Model

The unit cell structural model of Digne et al.^65^ was utilized. Unit cell dimensions (along crystallographic axes *a, b, c*) are *a* = 5.587 Å, *b* = 8.413 Å, and *c* = 8.068 Å. Selection of the crystallographic faces was based on considering γ-alumina nanoparticle morphology; among the most common crystal habits, the [110] facet comprises 70–83% of total exposed surface area, followed by the [100] facet, accounting for ∼17–30%.^65^ The crystallographic information file (CIF) for the unit cell model of Digne et al.^65^ was sourced from the template by Herráez, modified by Gutow.^66^ It is worth mentioning that the “[110]” and “[100]” terminations as described by Digne et al. are found at the [100] and [001] surfaces of their unit cell model, respectively. This is because the atomic lattice of the Digne et al. model is rotated by 45°, relative to a conventional face-centered-cubic (FCC) crystallographic unit cell. This rotation becomes apparent from the visual mismatch that occurs when attempting to find the [110] and [100] faces shown in literature diagrams^19,21−24^ at the corresponding conventional positions on the unit cell model.

**Figure 1:**
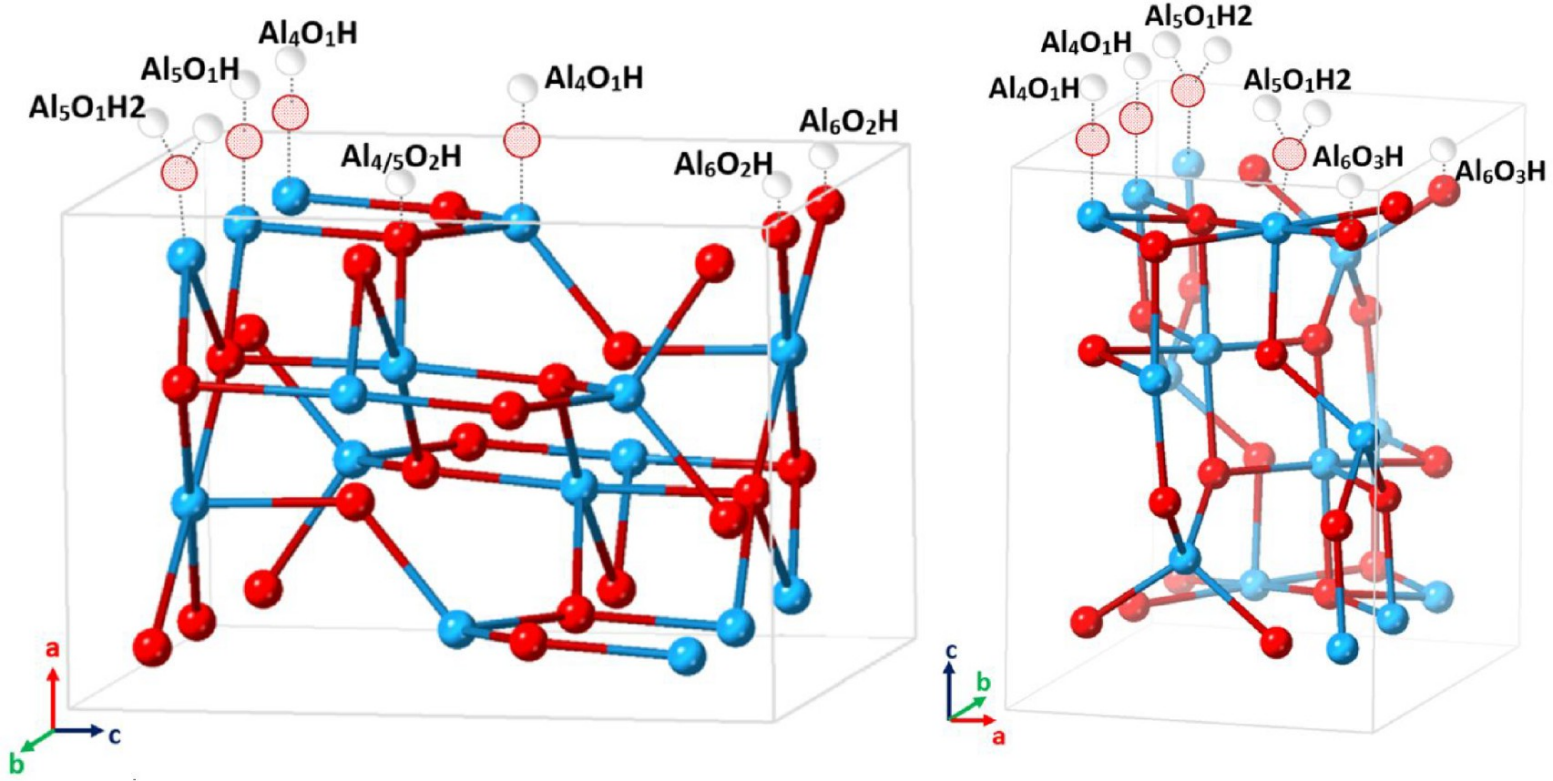
Schematics of the γ-alumina unit cell model, showing hydroxylation of the [110], left, and [100], right, crystallographic faces. As indicated by the crystallographic axes, the surfaces described as [110] and [100] are found at [100] and [001] positions of the unit cell model, respectively. The diagrams show single, complete unit cells, with unit cell boundaries (pale gray). Subscripts in the group labels signify ligancy of aluminum to oxygen, and of the hydroxyl group oxygen to aluminum atoms. For groups arising from surface oxygen atoms, for example, Al_6_O_3_H, the value for Al refers to the oxygen-coordination of aluminum atoms connected to the surface oxygen atom. Al = cyan, O = red (bold and pale, for oxygen atoms of the substrate and of attached groups, respectively), H = white.

In our work, the unit cell was replicated to create the γ-alumina substrates using the CrystalMaker^67^ software. The “Volume inspector” tool shows conventional lattice plane positions, indicated by Miller indices, with a sliding position scale. For the [110] γ-alumina surface ([100] on the unit cell model), the unit cell was replicated to 2a * 5b * 5c, yielding absolute dimensions of 11.17 * 42.065 * 40.34 Å. For the [100] γ-alumina surface ([001] on the unit cell model), the unit cell was replicated to 8a * 3b * 2c, yielding absolute dimensions of 44.696 * 25.239 * 16.136 Å. Because of periodic boundary conditions, the surfaces are effectively infinite in the *x* and *y* dimensions. Since substrates of similar dimensions have been utilized in prior classical MD studies for several interfacial systems,^38,39,68,69^ the simulation box dimensions used here are assumed large enough to minimize system size effects. To validate this assumption, simulations were repeated for a system of pure water as the aqueous phase, with a [100] substrate of doubled dimensions along the *Y* direction (yielding substrate dimensions of 44.696 * 50.478 * 16.136 Å). Results for water density profiles, ***ρ***(z), and residence times of interfacial water at this larger surface are consistent with the results shown in the main text; comparison is presented in SI Figure S1. Hydroxylation of the [110] and [100] surfaces was modeled following the ab initio study of Wakou et al.,^26^ yielding ∼10.3 and 12.8 OH groups per square nanometre for the [110] and [100] surfaces, respectively. In Figure 1 we report schematics for the hydroxyl group distribution on the two surfaces. The hydroxylation states considered are representative of acidic pH conditions, at which surface oxygen atoms are mostly protonated. Also visible in Figure 1 is the tricoordinated aluminum atom (coordinated to three oxygen atoms of the bulk structure), present as a metastable species exclusively on the prevalent [110] termination of γ- and δ-alumina particles. These atoms correspond to the sites of a known structural “defect” held responsible for the unique properties of “activated” (thermally pretreated) alumina, on which these species can be present as Lewis acid sites.^8,20^

Validation of the constructed surfaces was conducted via comparison with selected ab initio results for radial distribution functions.^26^ For the [110] and [100] surfaces, the literature ab initio results were obtained using 2 * 3 * 1 and 2 * 2 * 1 supercells, respectively, during 10 ps simulations at 308 ± 9 K (25.85–43.85 °C).^26^ Our MD results for [110] and [100] were obtained using 2 * 5 * 5 and 8 * 3 * 2 supercells, respectively, with 4 ns of data collection, at 293.15 K (20 °C). Despite the differences in simulated temperature and system sizes, and limitations of such comparison due to the different resolution of atomistic vs electronic structure calculations, MD results retain the main features of the ab initio data. This comparison is reported in SI Figure S2.

### 2.3 Force-Field Parameter Sets

#### 2.3.1 Water and Ion Pairs

The rigid simple point charge extended (SPC/E) water model^70^ was utilized to simulate water. O–H bond lengths and the H–O–H angle in each water molecule were maintained rigid using the SHAKE^71^ algorithm, as implemented in LAMMPS.

Force field parameters developed for use in conjunction with the SPC/E water model were applied to simulate ion pairs where possible. The widely used Joung-Cheatham model^72^ for sodium and chloride ions, parametrized for SPC/E water, was implemented, without polarizability. For barium nitrate, the parameters chosen for the nitrate ion^73^ have been utilized previously to study ion transport properties for aqueous (SPC/E) nitrate salts of sodium and potassium. Those parameters are implemented here with those of Mamatkulov et al.^74^ for the barium ion, developed to reproduce the solvation free energy of divalent ions with SPC/E water. For ammonium acetate, a parameter set^75^ incorporating the acetate ion, optimized to reproduce interactions with (TIP3P)^76^ water, and physiologically relevant cations, including ammonium, was utilized. Interaction energies are modeled using the Lennard–Jones and Coulomb potentials. Nonbonded interactions were truncated at 9 Å, as prescribed by the SPC/E water model.^70^ Mixed atom-type interaction potentials were calculated from self-interaction parameters, using Lorentz–Berthelot combining rules.^77,78^ In our recent work, it was demonstrated that the parametrizations implemented here reproduce experimental trends for bulk diffusion coefficients and viscosities of the respective aqueous solutions.^55^

#### 2.3.2 γ-Alumina

Bond, angle, charge, and pair-coefficient parameters for the surface atoms participating in hydroxyl groups (Al–O–H) were taken from ClayFF (Clay Force Field),^79^ which reproduces experimental trends for several mineral substrate–water interfaces.^80−82^ As implemented in ClayFF, surface hydroxyl groups were parametrized with the flexible SPC water model.^83^ With the exception of surface hydroxyl groups, atoms of the substrate are tethered to their initial position (as for most studies of similar systems^50,81,84^) with a spring force of 100 kcal/mol-Å. Doing this assumes that rotation and translation within the crystal structure is negligible within the simulation time frame, and that the initial orientation in which atoms of the substrate lattice are fixed has a minor impact on the properties of the hydration layer. Since our MD simulations reasonably replicate ab initio radial distribution functions (see SI Figure S2), this approximation is considered adequate. Quantifying how atomic vibrations within a solid substrate modulate properties of interfacial water requires force fields other than ClayFF. Geometric mixing rules were applied to calculate Lennard–Jones interaction parameters for unlike atoms (for example, between γ-alumina and the overlying aqueous phase).

### 2.4 Simulation Setup

**Figure 2:**
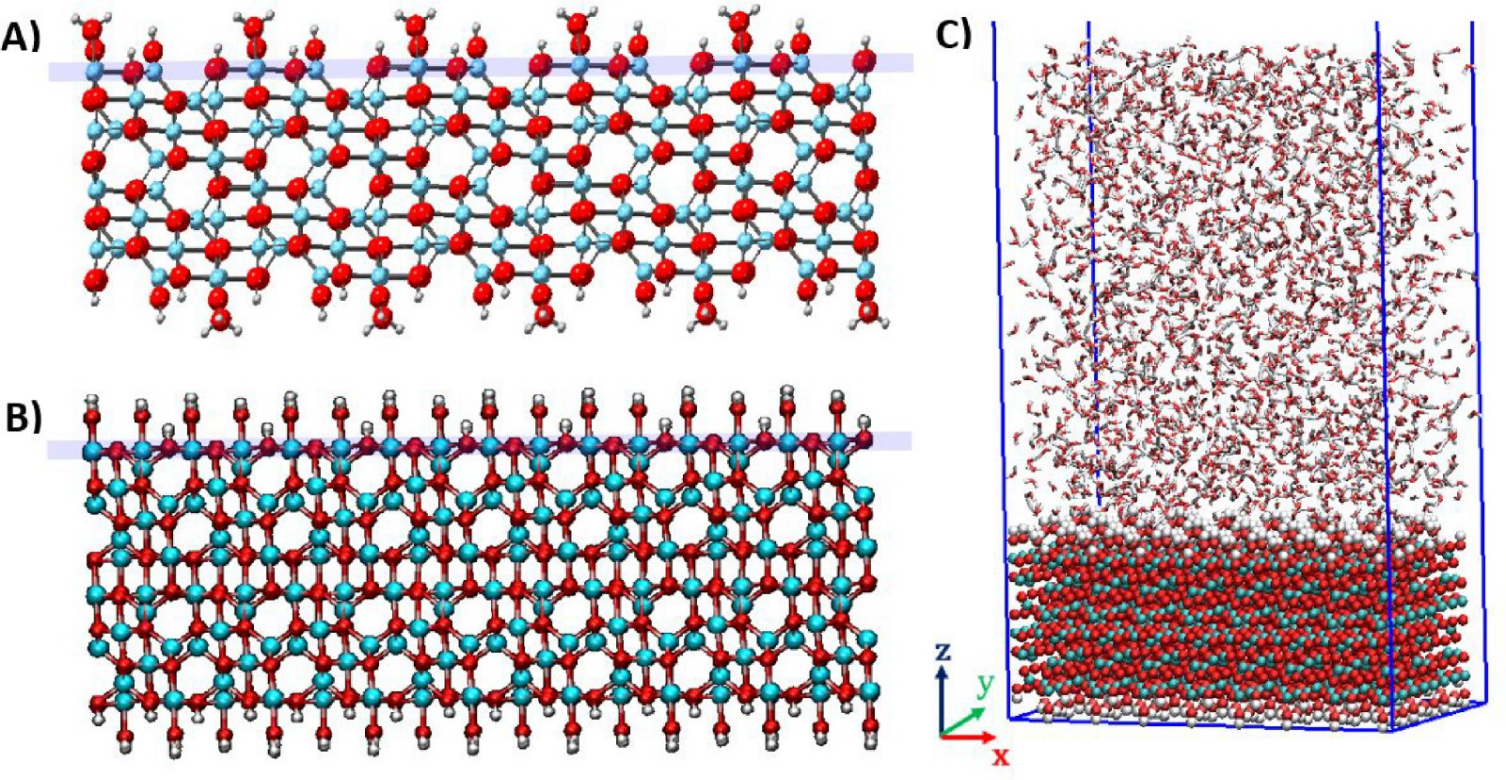
Side views of hydroxylated γ-alumina crystallographic surfaces (A) [110], (B) [100]. Lilac lines indicate the position of the “reference plane” for each surface, as referred to herein. Panel (C) shows a snapshot of initial system configuration (γ-alumina [100] and water). A film of fluid (water/aqueous solution) overlies the γ-alumina surface. Al = cyan, O = red, H = white. In panel (C), water = stick representation.

Simulation cells, with periodic boundaries along *x*, *y*, and *z* directions, were set up as shown in Figure 2. For ease of analysis, substrates were positioned with the crystallographic surface of interest parallel to the *x*–*y* plane. For all simulations of γ-alumina [110], simulation cells of 40.468 * 42.441 * 120.00 Å (*x*, *y*, *z*) were set up with 2523 water molecules overlying the substrate (substrate thickness of 11.17 Å). For all simulations of γ-alumina [100], simulation cells of 44.89 * 25.53 * 120.00 Å (*x*, *y*, *z*) were set up with 1912 water molecules overlying the substrate (substrate thickness of 16.136 Å).

The thickness of the liquid layer in our simulations varied between ∼50–53 and 58–60 Å, above the [110] and [100] surfaces respectively, depending on solvent composition. The space remaining in the *z* direction was left empty. The thickness of the liquid layer was chosen to ensure that its behavior was unaffected by periodic boundary conditions, and to allow sufficient room for “bulk” conditions to be established within the aqueous phase, located between the solid–liquid and liquid–vacuum interfaces. Adequacy of the film thickness was confirmed by ensuring that water density in the central region of the aqueous film reproduces the density of bulk liquid water at the thermodynamic conditions chosen for this study (see SI Figure S3).

For each of the two crystallographic terminations, five simulations were conducted with overlying films of: pure water, (1 molar) aqueous solutions of sodium chloride, barium acetate, ammonium acetate, and a 0.3 molar aqueous solution of barium nitrate. Ten simulations were conducted in total. The lower concentration of barium nitrate compared to the other aqueous solutions reflects its lower water solubility at ambient conditions.^85−87^

## 3 Results and Discussion

### 3.1 Hydration Structure

To elucidate the structure of pure liquid water at contact with [110] and [100] γ-alumina, we analyze our simulation trajectories to extract atomic density profiles perpendicular to the solid–liquid interface, and the preferential orientations and residence times of water molecules within the interfacial hydration layers. To reveal how the atomistic features of the two alumina surfaces yield differing interfacial hydration structures, we compute *x*–*y* plane density distributions, which visualize the arrangement of water molecules over both surfaces. These planar density distributions are then compared with the spatial distribution of surface features, including locations of OH groups, thus revealing regions where interfacial water preferentially accumulates.

#### 3.1.1 Density Profiles Perpendicular to γ-Alumina Surfaces

**Figure 3:**
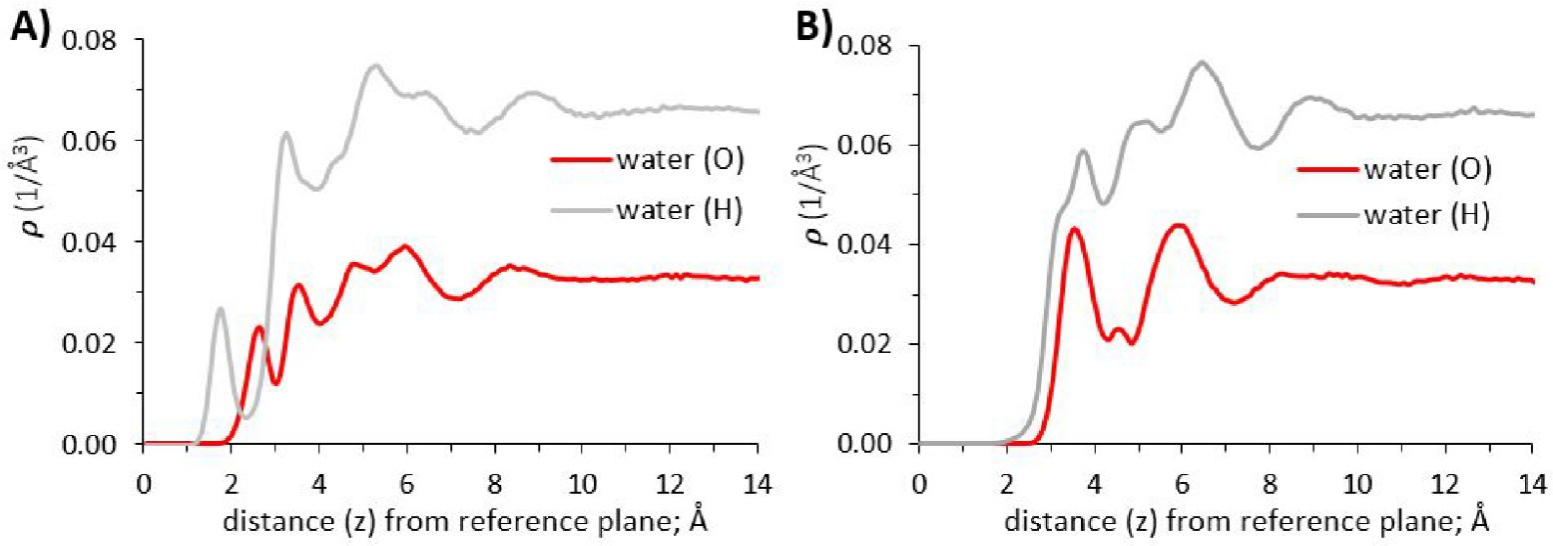
Atomic density profiles of oxygen and hydrogen atoms of water in the direction perpendicular to γ-alumina [110] and [100] surfaces; (A) and (B), respectively.

Atomic density profiles of water (oxygen and hydrogen atoms) were calculated as a function of distance (*z*) perpendicular to the γ-alumina surfaces. The reference plane (*z* = 0) for computing the vertical distance is the uppermost layer of aluminum atoms within the substrate (see Figure 2). In Figure 3 we compare the results obtained for the [110] and [100] γ-alumina terminations.

The density profile for water oxygen atoms above the [110] termination (Figure 3, panel A) shows the formation of four hydration layers (density peaks) near the surface, with two distinct layers located at 2.65 and 3.6 Å, respectively. Water density increases moving away from the interface into the third to fourth hydration layers, and then decreases to uniform bulk water density at distances ∼10 Å and further from the interface. These results suggest a depletion of hydration water at contact with the interface, relative to the bulk. Compared with results obtained on atomically smooth crystalline substrates in the literature,^36,68,88,89^ the depleted water density seen at the [110] interface is likely due to its atomic scale roughness^90^ (see Figure 2).

The first three peaks for the hydrogen atomic density profile (Figure 3, panel A) are located at 1.85, 3.35, and 5.45 Å, respectively. Considering peak positions and intensities of the oxygen and hydrogen density profiles together, the results just discussed reveal that the water molecules in the first hydration layer on [110] γ-alumina predominantly project one of their O–H bonds toward the interface. In the second layer, water molecules maintain both O–H bonds predominantly parallel to the surface, in some cases projecting one O–H bond away from the surface. The third peak of the hydrogen density profile, located at 5.45 Å from the surface, corresponds to the minima between the third and fourth peaks of the oxygen density profile. This is consistent with water molecules forming hydrogen bonds between the third and fourth hydration layers. Subsequent orientation analysis of interfacial water molecules (Section 3.1.2) and representative simulation snapshots (SI Figure S4) support the results just discussed.

Density profiles obtained for water oxygen atoms above the [100] γ-alumina (Figure 3, panel B), show two distinct hydration layers located at 3.65 and 5.95 Å, respectively, separated by a pronounced minimum at ∼4.65 Å from the reference plane. The first three peaks for the hydrogen density profiles (Figure 3, panel B) are located at 3.75, 5.15, and 6.55 Å. A shoulder is visible in the H density profile at 3.25 Å. These results reveal that within the first hydration layer (O density peak centered at 3.75 Å), water molecules mostly direct their O–H bonds either parallel to, or away from, the interface. For the second hydration layer, the oxygen density peak at 5.95 Å is accompanied by hydrogen peaks at 5.15 and 6.55 Å. The peak positions and intensities are consistent with water molecules directing O–H bonds both away from, and toward the interface; water molecules within the second hydration layer form hydrogen bonds with water molecules in both the first *and* third hydration layers, providing connectivity within the hydration structure of the [100] surface. Subsequent orientation analysis (Section 3.1.2) supports the results just discussed.

The density profiles of Figure 3 show that bulk-like water structure is recovered at 10 Å or further from the [100] surface, consistent with results obtained for the [110] surface. Overall, water accumulates near the [100] surface, while a somewhat depleted water population was observed near the [110] surface, albeit at closer contact. These results are consistent with (a) the surface density of OH groups, which is ∼24% larger on [100], compared to [110], and (b) the fact that the [100] surface is more atomically smooth than the [110], as seen in Figure 2.

#### 3.1.2 Molecular Orientation within Interfacial Hydration Layers

**Figure 4:**
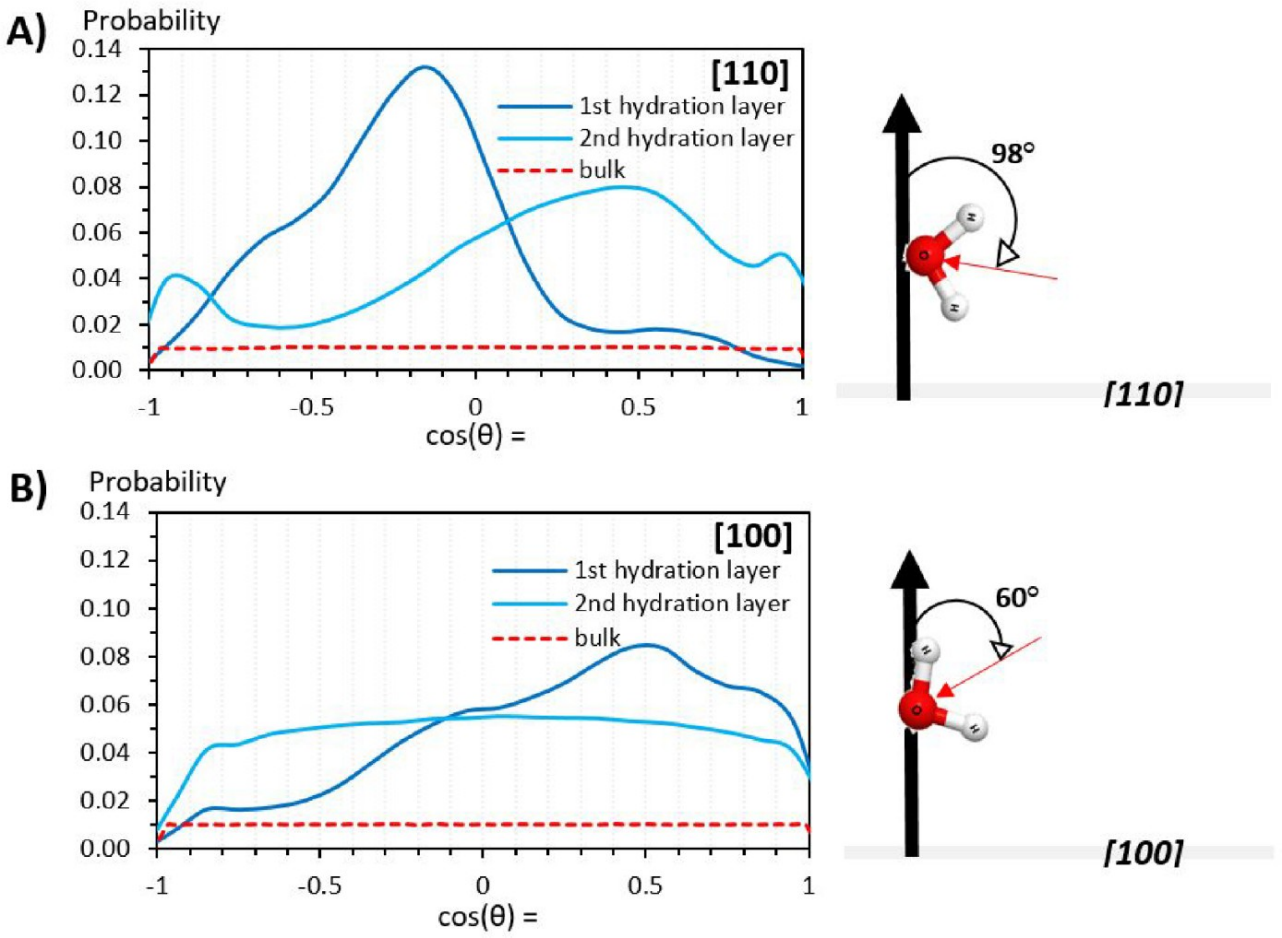
Orientation of water molecules at γ-alumina [110] and [100] surfaces; panels (A) and (B), respectively. The probability distributions are shown as the cosine of the angle between the vectors normal to the surface, and the dipole moment of water. Results for bulk water are the uniform distribution (dashed red line). Schematics on the right show the predominant orientation for water molecules in the first hydration layer of each surface. Red and black arrows show the water dipole moment and surface normal vectors, respectively.

Probability distributions of the angle (θ) formed between the water dipole moment and the vector perpendicular to the surface were computed for interfacial water molecules at the γ-alumina [110] and [100] terminations. Angles of 0° and 180° correspond to water molecules having *both* O–H bonds directed away from, and toward the surface, respectively. A 90° angle means that one O–H bond points away from the surface, and the other toward the surface. Figure 4 shows the probability distributions (***P***[cos(θ)]) for water molecules within first and second hydration layers of the γ-alumina [110] and [100] terminations, for pure water. Probability distributions obtained for bulk water are also shown for comparison. These show the expected uniform probability distribution.

Water molecules in the first two hydration layers of γ-alumina [110] show pronounced preferential orientations (Figure 4, panel A). By contrast, the [100] termination induces preferential water orientations only within its first hydration layer (Figure 4, panel B). These results are consistent with the density profiles discussed in Section 3.1.1.

Within the first hydration layer of the [110] surface, a pronounced peak at cos(θ) −0.15 indicates a strong likelihood for water molecules to form 98° angles between their dipoles and the surface normal. This implies one O–H bond pointing toward the surface, interacting with surface oxygen atoms, and the other almost parallel to the surface, forming hydrogen bonds with water molecules (oxygen atoms) in the second hydration layer. Water molecules in the latter adopt a wider range of orientations, but an overall directional shift of water O–H bonds away from the surface is observed. Water dipoles mostly form angles of 60° (cos^–1^(0.5)) with the surface normal. Also present are smaller populations of water molecules (cos(θ) = 0.95) and (cos(θ) = −0.95) with *both* O–H bonds directed either away from or toward the surface, respectively.

For the [100] surface, water molecules in the first hydration layer are orientated with predominantly 60° angles between their dipoles and the surface normal, seen from the peak at cos(θ) = 0.5; Figure 4 panel B. The peak intensities and the spread of the distribution appear broadly on par with those observed in the second hydration layer of [110]. This suggests a comparatively weaker influence of the [100] surface on its interfacial water population. This interpretation is further supported, going into the second hydration layer, by the wide spread of water molecule orientations. The water molecules show no predominant orientation, other than the decreased probability for the range of ∼148–180° (cos(θ) = −0.85 to −1); that is, both O–H bonds pointing toward the surface.

#### 3.1.3 Water Residence Times at Contact with γ-Alumina Surfaces

To complement the structural insights, we probe the dynamics of water within the interfacial regions, quantifying the residence autocorrelation function, *C*_R_(*t*), for interfacial water.^62,91^ The hydration layers of interest are of width ∼1A, centered at distances corresponding to the maxima in the atomic density profiles (Figure 3) of water oxygen atoms on the two γ-alumina terminations. To compute the average residence time of water molecules within a specified hydration layer, we extract ensemble averages for *C*_R_(*t*), which equates to 1 as long as a given molecule resides within the specified layer, and becomes 0 once the molecule leaves the layer. Should the molecule return to the layer of interest, its contribution to the residence autocorrelation function remains 0, following prior works.^62,91,92^ The slower the decay of *C*_R_(*t*) from 1 to 0, the longer, on average, water molecules reside within the hydration layer. When *C*_R_(*t*) is fitted with a single exponential function, the average residence time can be estimated as the time required for *C*_R_(*t*) to decay from 1 to 1/*e*. To provide an indication of statistical uncertainty for the computed averages, additional water residence time analyses were conducted at both γ-alumina terminations, starting from differing time origins. The results, reported in SI Figure S5, are qualitatively consistent with the plots discussed herein and provide an estimate on the uncertainty of the results discussed below.

**Figure 5:**
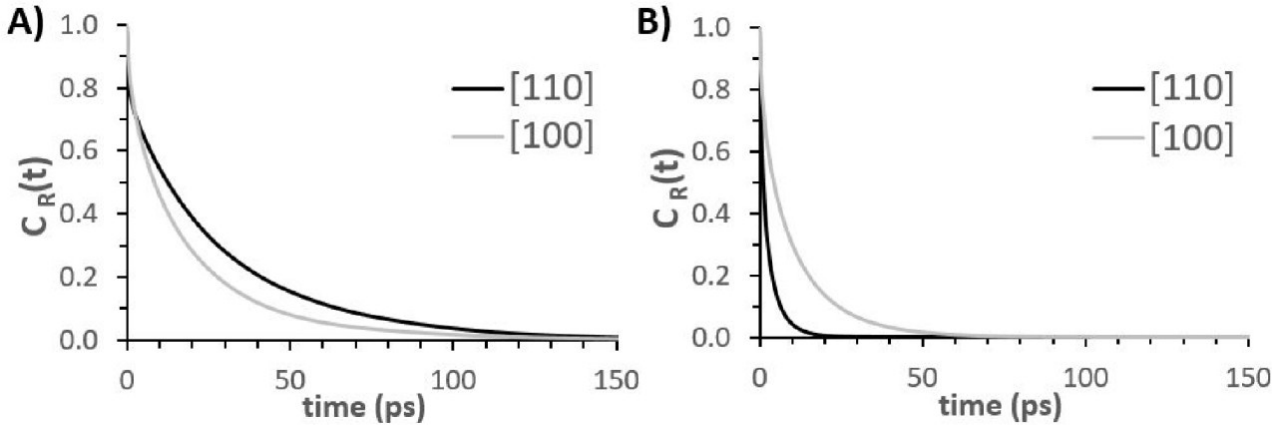
Residence autocorrelation functions for water (oxygen atoms) on γ-alumina [110] and [100] surfaces. Panel A: within the first interfacial hydration layer. Panel B: within the second interfacial hydration layer.

Results for *C*_R_(*t*) obtained for water molecules within first and second hydration layers on the [110] and [100] γ-alumina surfaces are shown in Figure 5. For both surfaces, water molecules reside in the first hydration layer longer than in the second, as commonly observed in the literature relevant to hydration water at solid surfaces. The results show that water is more mobile within the first hydration layer of [100], compared to [110]. This reveals that the γ-alumina [110] surface induces longer-lasting interactions with interfacial water molecules. This is likely due to the atomistic-scale roughness, more pronounced for the [110] termination, which increases closer-contact surface–water interactions. Within the second hydration layer, the surfaces induce the opposite effect, with water molecules residing for less time at the [110] surface, compared to [100]. This difference occurs since the second hydration layer on the [110] substrate is effectively an interstitial layer, as indicated by the thinner width of the corresponding peak in the (water) oxygen atomic density profile (see Figure 3). This interstitial layer provides connectivity between the water molecules of the first hydration layer, strongly adsorbed on the surface, and those further away; whereas within the more substantial second hydration layer of [100] γ-alumina, water resides for longer times before diffusing away.

#### 3.1.4 Relations between Surface Features and Interfacial Water Structure

To correlate the hydration structure with the surface features, density distributions of water within films identified by the first two interfacial hydration layers were computed. The results are referred to as planar (2-D) density distributions. Hydration layer positions were inferred from the first two peaks of the water (oxygen) atomic density profiles in Figure 3. The planar density distributions are computed within layers parallel to the surfaces. The thickness for each hydration layer is defined, from Figure 3, by the distance from the peak maxima to the minima either side. For the γ-alumina [110] surface, the first and second hydration layers reside between 1.95 to 2.95, and 2.95 to 4.05 Å from the reference plane, that is, thicknesses of 1 and 1.1 Å, respectively. For the γ-alumina [100] surface, the first and second hydration layers are identified from 2.75 to 4.35 and 4.95 to 7.15 Å, that is, of thicknesses of 1.6 and 2.2 Å, respectively.

Visual analysis of the planar density distributions identifies nanoscale regions within which water molecules accumulate. To relate these regions of preferential accumulation to the structure of the γ-alumina surfaces, maps illustrating the positions of surface features, for example, the OH groups, are superimposed onto the planar density distributions.

**Figure 6:**
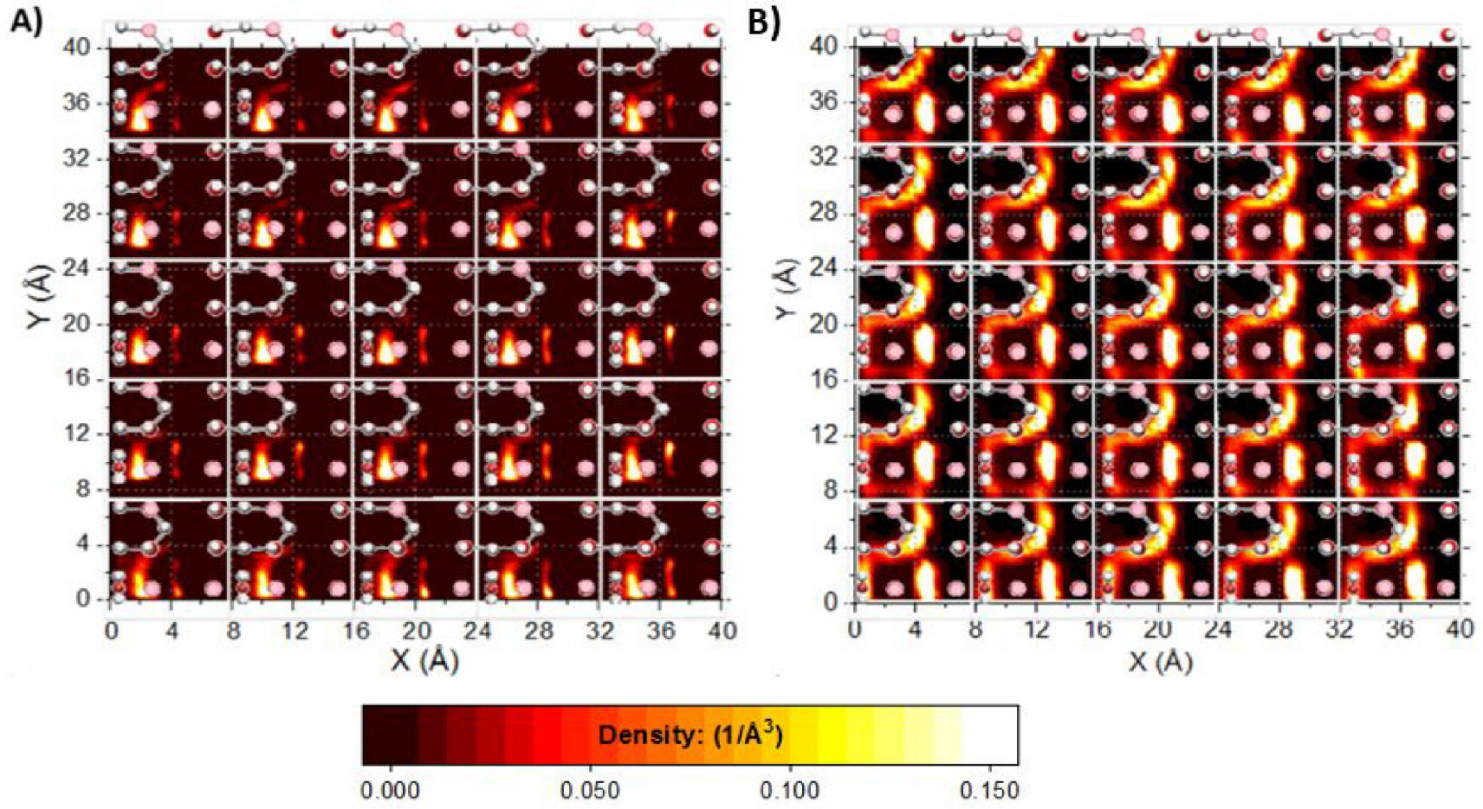
γ-Alumina [110] surface, superimposed onto planar density distribution graphs of interfacial water. Diagrams (A) and (B): water (oxygen) density distributions within the first and second interfacial hydration layers, respectively. Unit cells (white borderlines) are shown to aid interpretation. For clarity, only the surface atoms of γ-alumina [110] are shown. Surface groups are shown in initial configuration (bond vectors aligned normal to the surface) for clarity. Enlarged images, and overlays onto density distributions of water (hydrogen) are shown in SI Figure S6.

In Figure 6, the γ-alumina [110] surface is superimposed onto planar density distributions of water (oxygen) in the first and second hydration layers (panels A and B, respectively). In the first hydration layer, water preferentially adsorbs near a surface H_2_O group (Figure 6, Panel A). Simulation snapshots (SI Figure S4) show water molecules at this site directing one of their O–H bonds downward, H-bonding with a surface oxygen atom located adjacent to the H_2_O surface group. The other water O–H bond links to water molecules in the second hydration layer, and in doing so, bridges a structural cavity feature of the unit cell surface. In the second hydration layer, water oxygen atoms interact with H_2_O surface groups across the unit cell boundary. In the second hydration layer (Figure 6, Panel B) a pattern of bright spots above the structural “cavity” feature on the substrate indicates where water molecules accumulate to bridge over the surface cavity. A second, arc-like, density distribution pattern (Figure 6, Panel B) traces the path of water interaction with other alumina surface groups, for example, Al_4_O_1_H, shown in representative simulation snapshots (SI Figure S6a,b).

**Figure 7:**
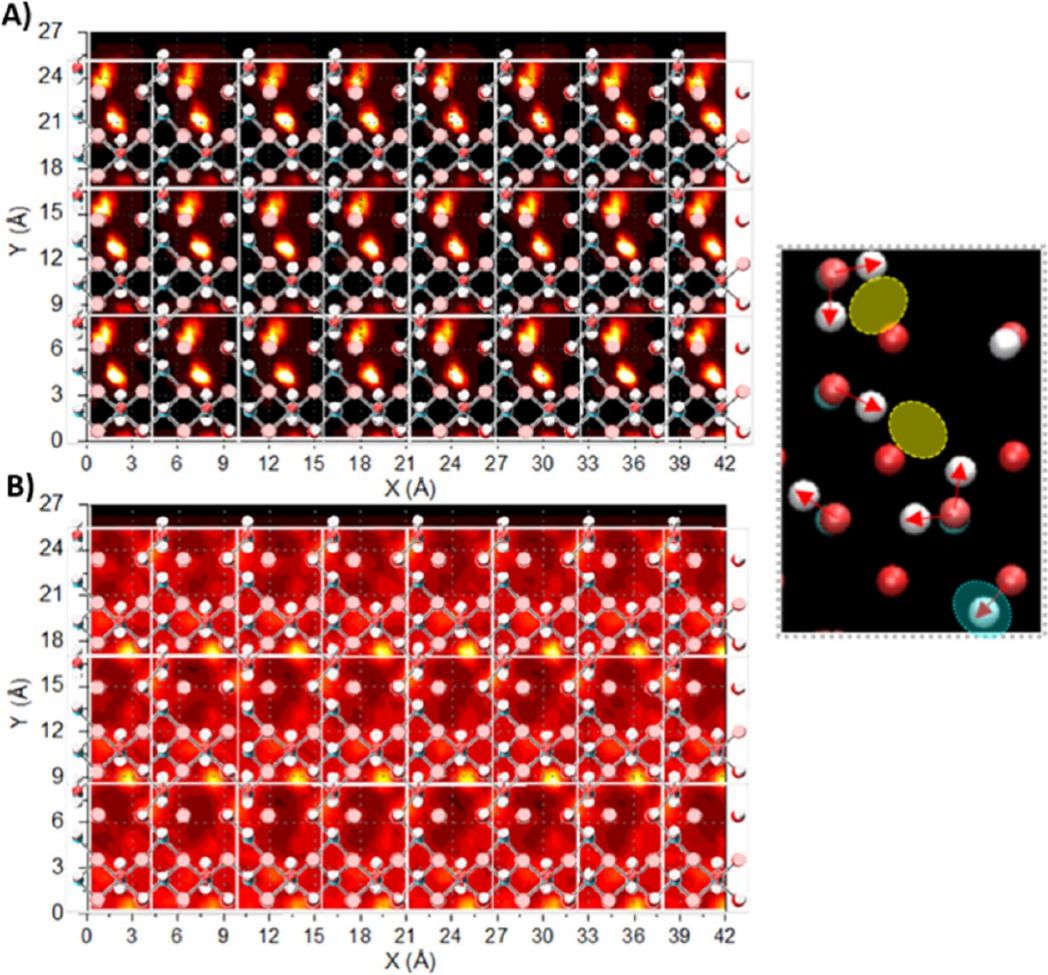
γ-Alumina [100] surface, superimposed onto planar density distributions of interfacial water. Diagrams (A) and (B): water (oxygen) density distributions within the first and second interfacial hydration layers above the γ-alumina [100] surface, respectively. For clarity, only the surface atoms of γ-alumina [100] are shown. Unit cells (white borderlines) are shown to aid interpretation. For clarity, surface groups are shown in initial configuration (bond vectors aligned normal to the surface). The simulation snapshot shows a single unit cell surface (aerial view) with representative orientations of surface groups (indicated with red arrows). Yellow and blue highlights correspond to the regions of high OW density in the first and second hydration layers. Enlarged images, and overlays onto density distributions of water (hydrogen) are shown in SI Figure S7.

Figure 7 shows the planar density distributions of water oxygen and hydrogen atoms within the two interfacial hydration layers of the [100] surface. Water is more ordered within the first hydration layer compared to the second, as can be seen from the more clearly defined distributions of high density. One pattern of high-density regions within the first hydration layer correlates with the hydrogens of surface Al_5_O_1_H_2_ groups (one of the two present per unit cell). A second pattern of high density, also within the first hydration layer, correlates to a potential “bridging zone” between hydrogens of the second Al_5_O_1_H_2_ group and one of the Al_4_O_1_H groups. In this context, a small proportion of water molecule O–H bonds within the first hydration layer are directed toward the interface, while the majority lie predominantly parallel or away from it, consistent with the results shown in Figure 4. These two orientations correlate with the two patterns just described, respectively.

Within the second hydration layer above γ-alumina [100], some high-density regions of water (oxygen) are observed, corresponding to the location of surface Al_6_O_3_H groups. Nevertheless, the planar distribution of water is rather diffuse in the second hydration layer. This contrasts with results on γ-alumina [110], where structural perturbations of water extend into the second hydration layer.

Compared to γ-alumina [110], the [100] surface has a higher surface density of OH groups (12.9 compared to 10.3 OH/nm^2^) and, in the model used here, hosts two H_2_O surface groups per unit cell, compared to one for [110].

Contrasting with the more closely packed features of [100], the [110] surface displays more heterogeneity, in terms of local surface features; intimately neighboring areas with and without surface OH groups. This yields stronger interactions and further-reaching perturbation effects on water structuring. This observation agrees with findings from Wakou et al.,^22^ in that, compared to [100], the [110] surface favors local structuring of water and solvation of its μ1-OH and μ1-H_2_O groups. By contrast, on [100], a stronger H-bond network among μ1-OH and μ1-H_2_O groups reduces water-surface interaction. The results illustrate how a combination of the surface density and spatial distribution of OH groups, and the spatial distribution of atomistic-scale roughness of a substrate, directly affect the structure of interfacial water.

### 3.2 Salt-Specific Perturbations of Hydration Structure and Dynamics

While the results above quantify how substrate characteristics affect the properties of pure water in contact with γ-alumina, this section explores how different salts disrupt the properties of interfacial water. A variety of phenomena can be expected. For example, Xu et al.^93^ showed that, even though the structure of interfacial water on the [001] termination of corundum (α-Al_2_O_3_) changes little when the pH ranges from 5 to 9, the presence of arsenate causes substantial restructuring, which suggests the adsorption of solutes can have stronger effects than changes in surface charge density. Wang et al.^94^ demonstrated that, because structurally ordered interfacial water facilitates hydrogen evolution reactions on atomically flat Pd, the presence of Na^+^ ions indirectly affects the reaction rates, via perturbing the structure of the hydration water. Cao and Netz^95^ demonstrated that a combination of water orientation, hydrogen-bond network, surface features, and presence of salt ions lead to anomalous electro-kinetic effects within graphitic pores. Baryiames et al.^96^ demonstrated that hydrogen bond populations measured for water molecules at the interface with oil in the presence of nonionic surfactants changes little upon addition of Na^+^ and Ca^2+^ ions, yet the dynamics of interfacial water molecules were significantly more sluggish in the presence of the ions.

MD simulations are employed here to help us understand the molecular mechanisms responsible for changes in relative orientation, hydrogen-bond density, and mobility of interfacial water upon the addition of ion pairs that differ in size, shape, and charge density.

#### 3.2.1 Density Profiles Perpendicular to γ-Alumina Surfaces

**Figure 8:**
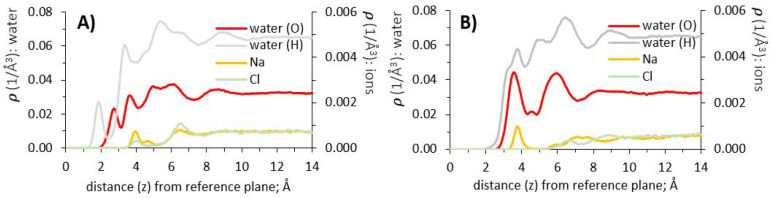
Atomic density profiles of water and sodium chloride (1 molar aqueous solution), at surfaces of γ-alumina [110] and [100]; (A) and (B), respectively.

**Figure 9:**
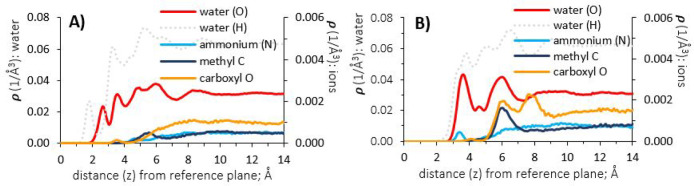
Atomic density profiles of water and ammonium acetate (1 molar aqueous solution), at surfaces of γ-alumina [110] and [100]; (A) and (B), respectively.

**Figure 10:**
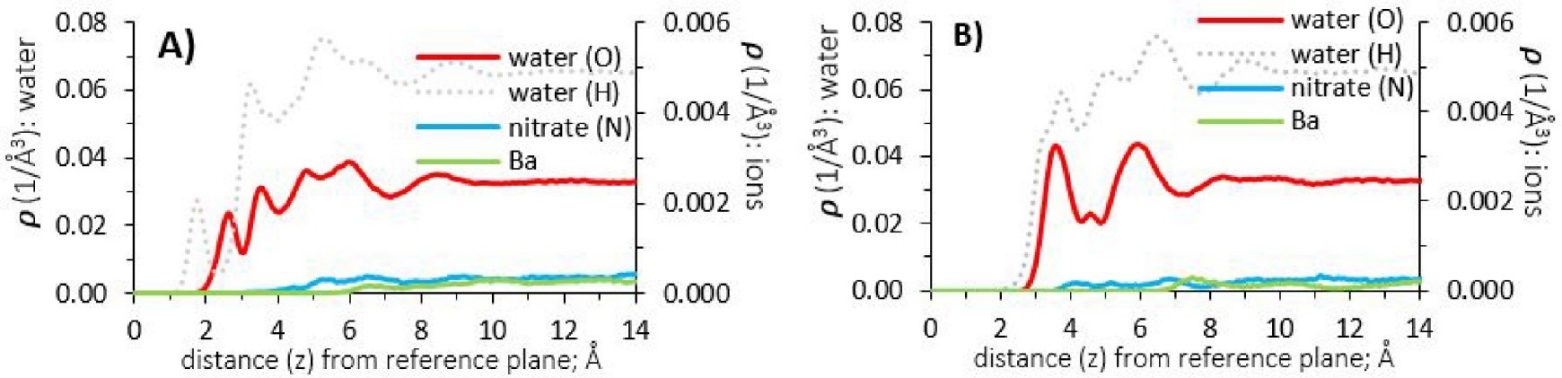
Atomic density profiles of water and barium nitrate (0.3 molar aqueous solution), at surfaces of γ-alumina [110] and [100]; (A) and (B), respectively.

**Figure 11:**
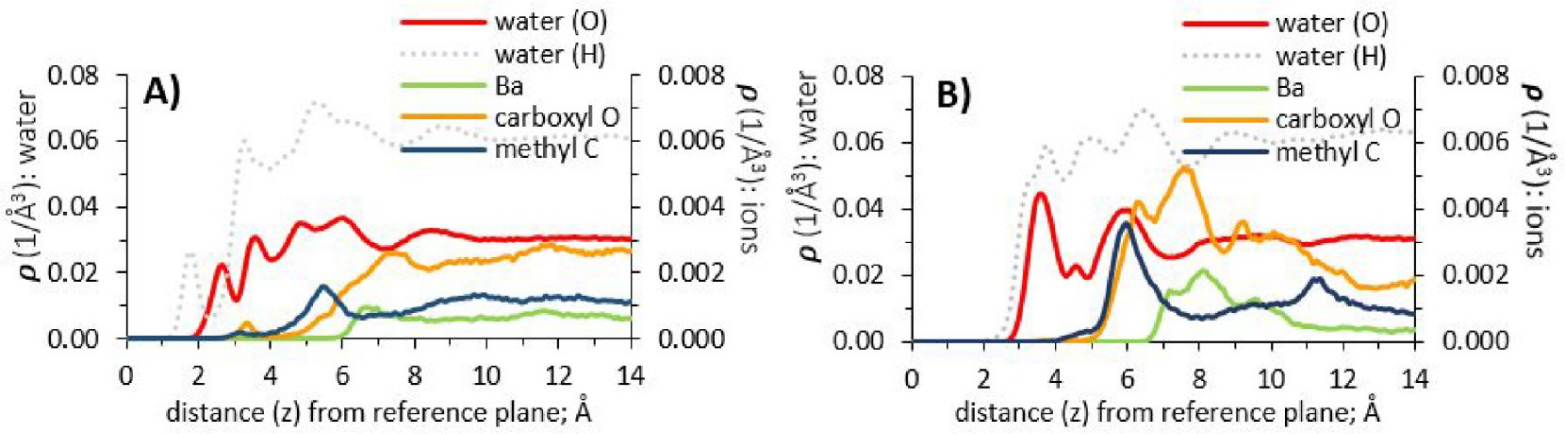
Atomic density profiles of water and barium acetate (1 molar aqueous solution), at surfaces of γ-alumina [110] and [100]; (A) and (B), respectively.

Upon dissolution of ion pairs, the atomic density profiles for O and H atoms of water in the direction perpendicular to the γ-alumina [110] and [100] surfaces do not change much compared to those reported for pure water, beside a minimally reduced density away from the immediate interfacial zone. These results are shown in SI Figure S8. To identify where ions accumulate near the surfaces of interest, Figures 8–11 show atomic density profiles obtained for sodium chloride, ammonium acetate, barium nitrate, and barium acetate. Each figure also shows water O and H density profiles, for comparison, and displays results for the [110] and [100] terminations.

The density profile for sodium on the [110] surface (Figure 8, panel A) shows a first peak at 4.05 Å, within the second hydration layer, and a smaller shoulder, at 4.65 Å, moving into the third hydration layer. This indicates that Na^+^ ions are excluded from the hydration structure at close contact with the [110] substrate. This could be a result of the atomically “rough” surface, combined with the tendency of Na^+^ ions to maintain their own hydration shell. The next density peak for Na^+^ ions is observed at 6.55 Å, where our results show co-residence of sodium and chloride ions (Figure 8, panel A) within the fourth hydration layer. Chloride ions interact with water hydrogens, as shown by the alignment of the chloride density peak with the shoulder of the water hydrogen density profile, also located at 6.55 Å from the surface. At this distance from the interface, both sodium and chloride ions maintain energetically favorable hydration shells and also interact with each other to minimize electrostatic interactions. For both ions, our results show uniform density profiles at distances greater than ∼10 Å, where water density profiles approach bulk values.

Considering sodium chloride on [100] (Figure 8, panel B), the density profiles show a peak of Na^+^ ions at 3.85 Å, within the first hydration layer, while chloride ions emerge beyond the second hydration layer. This shows that sodium chloride dissociates in the proximity of [100] more effectively than near the [110] surface, when comparing the density profiles obtained from these two surfaces (Figure 8).

Results obtained for ammonium acetate on γ-alumina [110] (Figure 9, panel A) indicate that these ions avoid the immediate interfacial region. Uniform density profiles are obtained beyond the hydration layers, where water density becomes uniform, suggesting that the interfacial water at the [110] surface is not able to accommodate the optimal hydration configuration for the ammonium and acetate ions, which are consequently repelled from the surface.

Figure 9 (panel B) shows the density profiles for ammonium acetate on the [100] surface. The density profile for ammonium ions shows a small peak at 3.45 Å, within the first density peak of water oxygen atoms, while close enough to the substrate to also interact with oxygen atoms of surface groups. Within the second hydration layer, the density peak of acetate ion methyl groups at 5.75 Å interacts with water oxygens. The double peaks of carboxyl oxygen atoms, at 6.15 and 7.75 Å, indicate that one carboxyl oxygen points toward, and one away from the interface, straddling the third peak of the water hydrogen density profile. Comparing the [110] and [100] surfaces, for the density profiles of sodium chloride and ammonium acetate (Figures 8 and 9), the hydration structure at the [100] surface induces a separation of cations and anions between the first and second hydration layers, respectively.

In terms of ion exclusion from the hydration structure of the [110] surface, density profiles for barium nitrate (Figure 10, panel A) show similar trends to ammonium acetate, although present at lower concentration (0.3M) due to its lower water solubility. The barium cations reside further from the [110] surface than their nitrate anion counterpart; this observation also applies for barium acetate (Figure 11, panel A). This is likely due to the large ionic radius of barium and its 8-fold water coordination; difficult to incorporate within the water structure of the near-interface. Smaller monovalent ions do not encounter such combination of steric and hydration effects, as shown by the atomic density profiles of sodium chloride and ammonium acetate, previously discussed.

Density profiles of barium nitrate on the [100] surface (Figure 10, panel B) show nitrate ions bordering the first hydration layer, that is, slightly closer to the surface, while barium ions accumulate slightly further from the surface (beyond 7 Å), compared to results obtained on the [110] termination.

The density profiles for barium acetate on γ-alumina [100] (Figure 11, panel B) are consistent with the interaction of acetate ion methyl groups with (water) oxygen in the second interfacial hydration layer (at 6.05 Å from the interface). A broad density peak of barium ions, located at ∼8.25 Å, is encompassed within an even broader region of acetate carboxyl groups with peak density at ∼7.75 Å, a likely result of some degree of ion pairing. For the barium ion, previously discussed size-related effects consign its residence out of the first and second hydration layers to beyond 6 Å from the interface, similar to results obtained on the [110] surface.

The results discussed in this section indicate that ions are more easily accommodated within the interfacial hydration structure of γ-alumina [100], compared to the [110] surface. This is illustrated for the [100] surface by the stronger correlation of ion density peak positions to the water O and H density profiles, and the closer proximity of ions to the interface, compared to [110].

#### 3.2.2 Effect of Salts on Interfacial Water Structure

**Figure 12:**
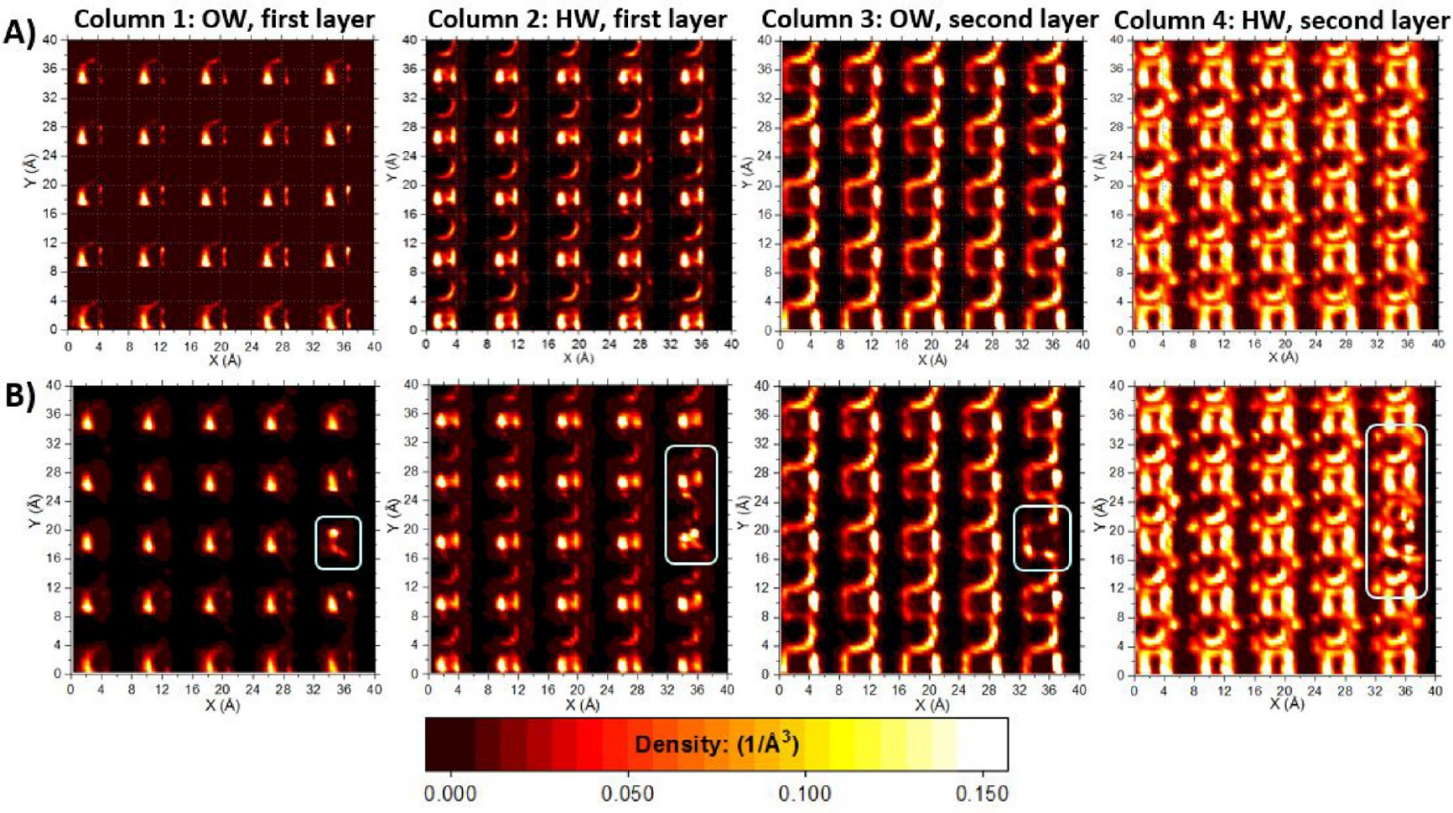
Planar density distributions of water over the γ-alumina [110] surface, for pure water (Row A) and 1 molar aqueous solution of barium acetate (Row B). Columns 1 and 2: first hydration layer, water oxygen and hydrogen (OW and HW), respectively. Columns 3 and 4: second hydration layer, water oxygen and hydrogen (OW and HW), respectively. The scale bar is applicable to all graphs.

Complete results of planar density distributions at the [110] surface, for pure water and aqueous salt solutions, are provided in SI Figure S9. All salts considered visibly “diffuse” surface density distributions of water oxygen and hydrogen atoms within the first hydration layer. For barium acetate, localized hydration structure distortions are seen within both the first and second hydration layers, as indicated in Figure 12 (panel B, blue outlines). Comparison with planar density distributions of the ions (SI Figure S10) show that these distortions occur in the vicinity of acetate ion carboxyl groups, which—as seen from the corresponding density peak in Figure 11 (panel A)—accumulate between the first and second hydration layers. Of the cations considered, the highest concentrations closest to the [110] interface are attained by sodium, within the second hydration layer; the planar density distributions of sodium are shown in Figure S10.

**Figure 13:**
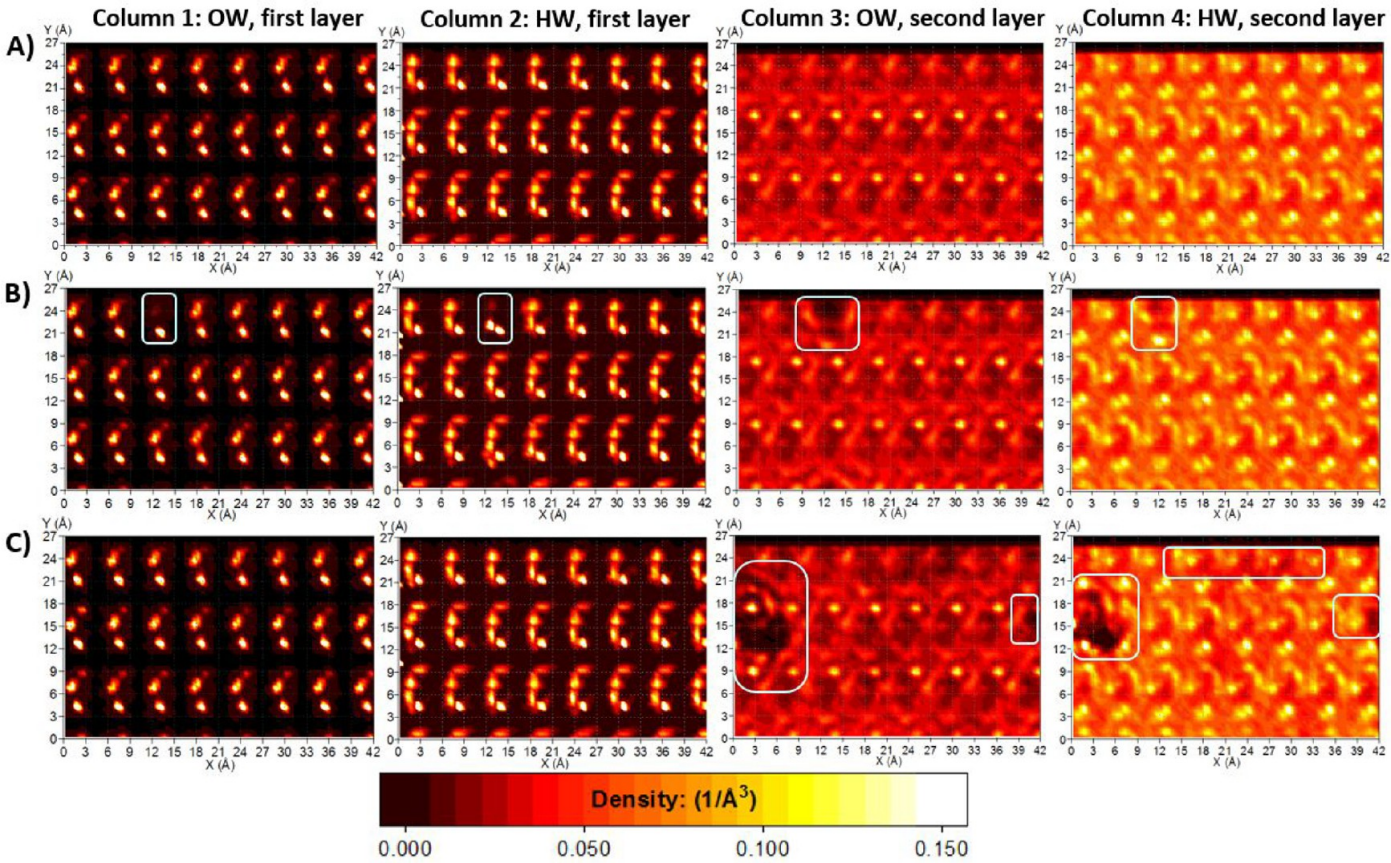
Planar density distributions of water over the γ-alumina [100] surface, for pure water (Row A), 0.3 molar aqueous solution of barium nitrate (Row B) and 1 molar aqueous solution of barium acetate (Row C). Columns 1 and 2: first hydration layer, water oxygen and hydrogen (OW and HW), respectively. Columns 3 and 4: second hydration layer, water oxygen and hydrogen atoms (OW and HW), respectively. The scale bar is applicable to all graphs.

On the [100] surface, notable distortions of the hydration layers are observed in the presence of barium nitrate and barium acetate, corresponding to ions accumulation sites near the interface, shown in SI Figure S11. For barium nitrate, localized disruptions of interfacial water distribution within the first and second hydration layers (Figure 13, row B, blue outline) result from nitrate anion adsorption within the interfacial region. Outlined in Figure 13 (row C), such effects also occur with barium acetate, but only in the second hydration layer, as expected, based on the atomic density profile peak positions (Figure 11, panel B) for barium and acetate ions. The hydration structure interference outlined in Figure 13, row C, where hydration water is displaced, corresponds to a region of complex ion association (Ba - acetate carboxyl groups) in the second hydration layer, detailed with simulation snapshots in SI Figure S12. Some effects, less disruptive than those just discussed, are also seen for sodium chloride and ammonium acetate, shown in SI Figure S13. For sodium chloride (Figure S13; row B, column 2), a weak localized distortion of water hydrogen structuring within the first hydration layer corresponds to an adsorption site of sodium (SI Figure S11, Panel A). For ammonium acetate (Figure S13; row C, column 4) within the second interfacial hydration layer, density values of water hydrogen appear to decrease in the vicinity of acetate anion methyl groups (SI Figure S11, Panel B).

**Figure 14:**
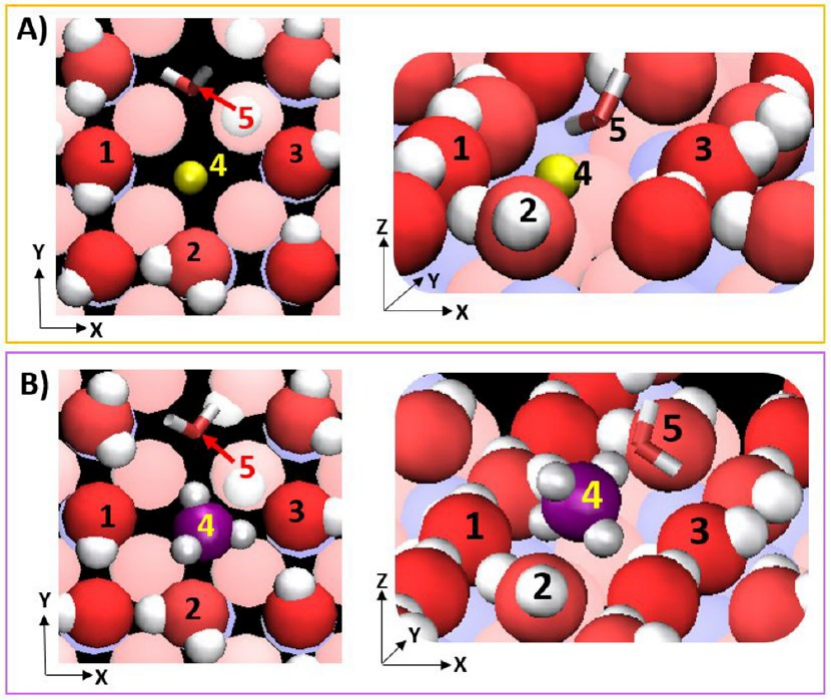
Simulation snapshots showing interactions of sodium and ammonium ions (label 4 in Panels A and B, respectively) at the γ-alumina [100] surface. Nearest-neighbor alumina surface groups Al_4_O_1_H (labels 1, 3) and Al_5_O_1_H_2_ (label 2) are indicated. For both ions, a water molecule (stick representation, label 5) from within the first hydration layer stabilizes the adsorbed configuration.

Comparing results for the two surfaces considered, the diminished water-surface interaction on γ-alumina [100], discussed in Section 3.1, facilitates closer association of ions with this surface, allowing more pronounced ion-specific effects on the interfacial water structure, including regions from where water molecules are displaced. Of the ions considered here, sodium and ammonium cations have the strongest affinity for the [100] interface. In the simulated trajectories, sodium ions can associate with a single adsorption site for up to ∼700 ps, interacting with oxygen atoms from three alumina surface groups; two Al_4_O_1_H groups, and an Al_5_O_1_H_2_ group, as well one water molecule that appears to “stabilize” the configuration, as shown in Figure 14, Panel A. Further detail and close-ups are shown in SI Figure S14. These same sites provide the preferred adsorption locations for ammonium ions (Figure 14, Panel B), shown with further detail in SI Figure S15.

**Figure 15:**
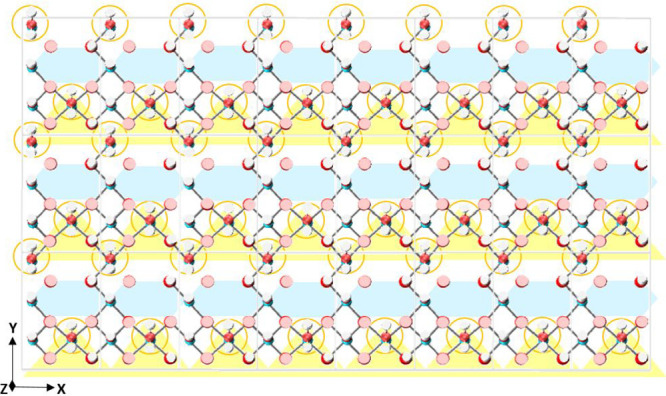
[100] γ-alumina surface. For clarity, only surface atoms are shown (atoms of the reference plane and surface groups). Unit cells (faint gray borderlines) are shown to aid interpretation. White = hydrogen, bright red = oxygen atoms part of a surface group, pale red = surface oxygen atoms. On the basis of observations from simulations, the surface is color-coded to show adsorption sites for cations and anions present within the first hydration layer. Regions of cation adsorption are shaded blue. For anions, the sites, between triads of H_2_O surface-groups (orange circles), are shaded yellow. Note that the surface is periodic in *x* and *y* directions.

Adsorption of anions is also observed at the [100] surface, although for shorter duration compared to the cations, suggesting weaker association. Of the anions considered, nitrate shows strongest adsorption at the [100] interface, at sites situated between three Al_5_O_1_H_2_ groups. These are also the preferred adsorption sites for chloride ions, where they can reside for up to ∼380 ps in our simulations. Simulation snapshots of nitrate and chloride ion interactions at the interface are provided in SI Figures S16 and S17, respectively. Our observations are visually summarized in Figure 15, where we demarcate preferential adsorption locations for cations and anions at the [100] surface (first hydration layer). Sodium and ammonium adsorb closest to the interface (see Figures 8 and 9, panel B), in regions between oxygen atoms of the reference plane, while nitrate and chloride ions adsorb between triads of H_2_O surface groups, further away from the interface.

#### 3.2.3 Interfacial Water Residence Times, in the Presence of Salts

**Figure 16:**
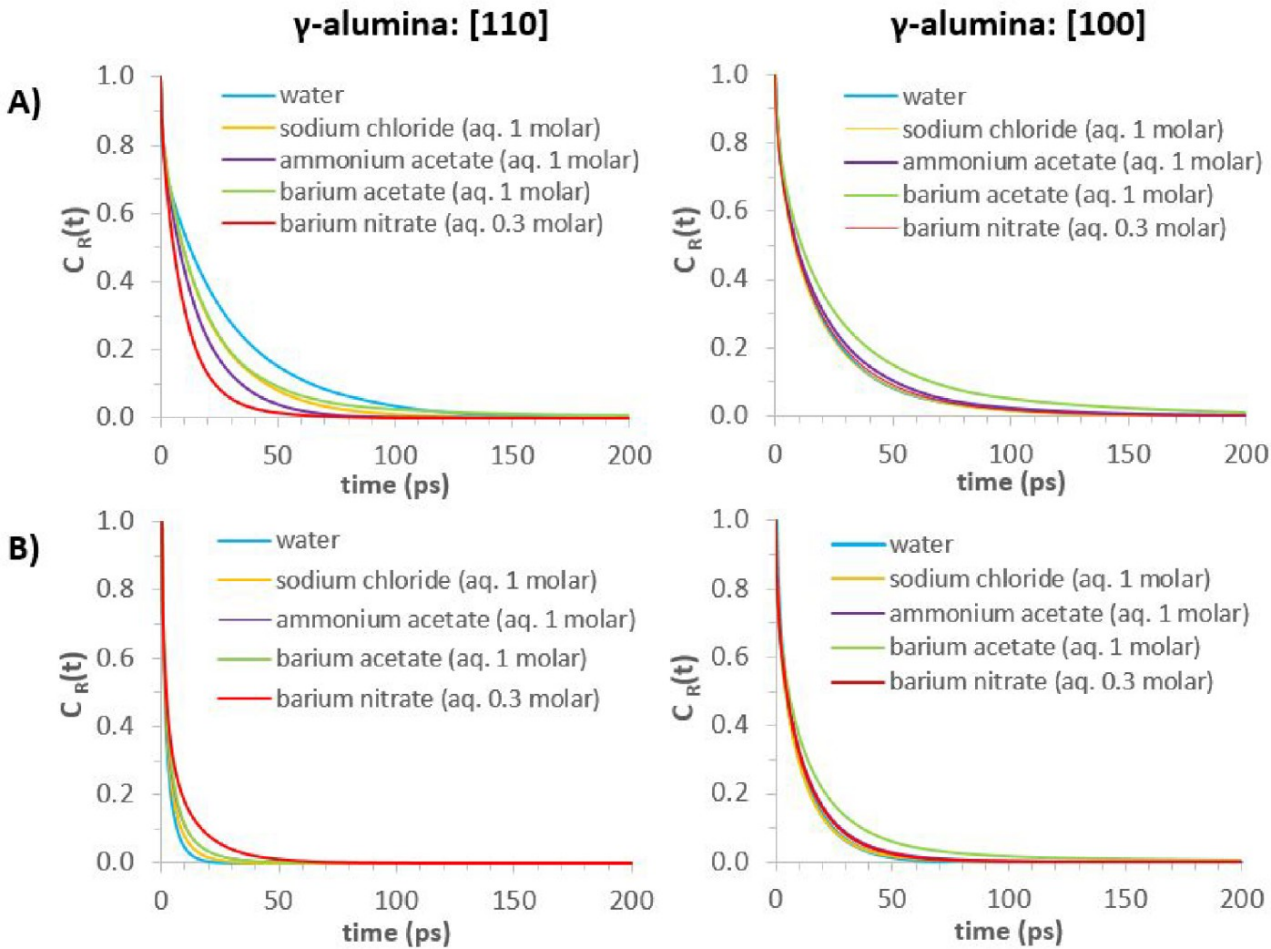
Residence autocorrelation functions—*C*_R_(*t*)—for water in the first and second hydration layers of γ-alumina [110] and [100] surfaces. Aqueous phase compositions indicated in graph legends. Column 1: [110] γ-alumina. Column 2: [100] γ-alumina. Rows (A) and (B): first and second hydration layers, respectively.

Results for residence autocorrelation function (Figure 16) show that within the first hydration layer on γ-alumina [110], all salts accelerate the dynamics of interfacial water to varying degrees, compared to pure water, but have the opposite effect on the second hydration layer. It is likely that ions accelerate water dynamics of the first hydration layer through ion–water interactions competing with surface–water interactions. For barium nitrate, this competition is seen with nitrate ions near the preferred adsorption sites of water at the [110] surface, as shown by the simulation snapshots in SI Figure S18. In the second hydration layer, the observed deceleration of water dynamics likely reflects the residence time of water within the solvation shells of ions, which accumulate at this distance from the surface; that is, the results are consistent with a stronghold shift from surface–water to ion–water interactions.

Contrasting with the results just discussed, water residence times on the [100] surface are not significantly affected by salts (Figure 16), except for barium acetate, which decelerates water dynamics in both the first and second hydration layers. Comparing Figures 12 and 13, ion-induced disruption of hydration structure is evident in the form of localized water “displacement” from γ-alumina [100], facilitated by its weaker surface–water interaction. For the [110] surface, the “distortion” of water density distributions (Figures 12) suggests that ion–water interactions compete with surface–water interaction, with resulting effects on water residence times at the interface. For the two γ-alumina surfaces, a ranking of interaction affinities can be proposed to explain the differing structure and dynamics of interfacial water; surface–water > ion–water > ion–surface, for [110] γ-alumina, and ion–surface > ion–water > surface–water for [100] γ-alumina.

#### 3.2.4 Influence of Salts on Interfacial Water Orientation

**Figure 17:**
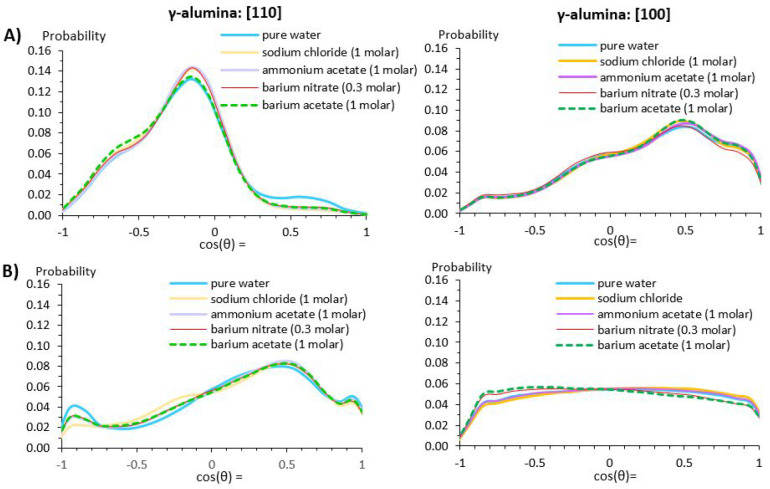
Orientation of water molecules at γ-alumina [110] and [100] surfaces; rows (A) and (B) show orientations within first and second interfacial hydration layers, respectively. The results show probability distributions for the cosine of the angle between the surface normal vector and the net dipole moment of water.

The probability distribution of the cosine of the angle (θ) between the water dipole moment and the surface normal is computed for interfacial water molecules in the presence of ion pairs. The results are shown in Figure 17 for water molecules within the first (Row A) and second (Row B) hydration layers of the γ-alumina [110] and [100] terminations. Interfacial water on the [110] surface is more strongly affected by the presence of ion pairs. In the first hydration layer, the salts reduce the probability of water dipoles at ∼37°–70° to the surface normal, while the likelihood of the ∼100° angle is increased. Considered together, this implies a net effect of more water O–H bonds pointing toward the surface, overall. In the second hydration layer, the opposite effect is seen, via the probability reduction in the range of 143–180° (between cos(θ) = −0.8 to −1).

At γ-alumina [100], the presence of salts has scarce effect on water orientation in the first hydration layer. By the second hydration layer, the lack of a dominant orientation indicates diminished influence of the surface; only the divalent barium nitrate and barium acetate salts mildly increase the probability for the range of angles ∼148–180° (cos(θ) = −0.85 to −1); that is, water molecules with both O–H bonds pointing toward the surface. When interpreting these results, it should be noted that the effects seen are rather small due to the lower ion concentration at the interface compared to the bulk.

#### 3.2.5 Salts Effects on the Hydrogen-Bond Network

**Figure 18:**
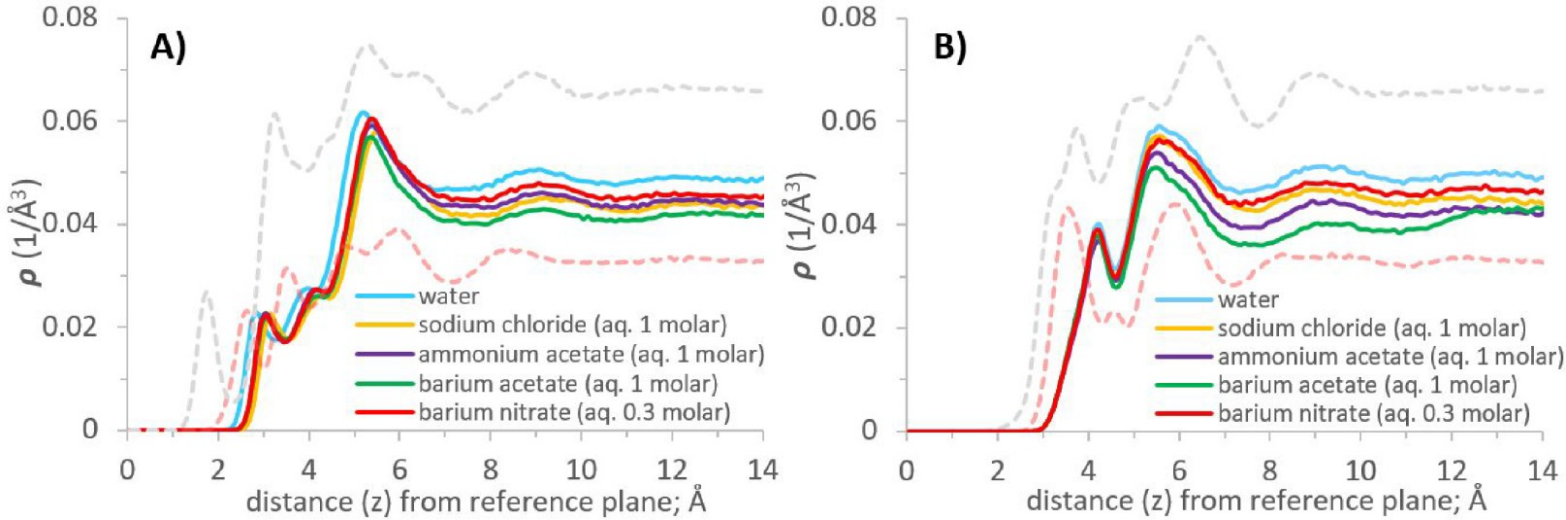
Density profiles of water–water HBs along the distance perpendicular to the (A) [110], and (B) [100] γ-alumina surfaces, respectively. Aqueous phase compositions indicated in graph legends. For comparison, atomic density profiles of water O and H are also included, represented by the red and gray dashed lines, respectively.

To further quantify the effects of salts on interfacial water, hydrogen bond (HB) density profiles were calculated for water molecules, as a function of the distance (*z*) perpendicular to the surfaces. Implementing the geometric criterion defined by Marti,^97^ two water molecules are considered hydrogen-bonded if the distance between a hydrogen atom in one water molecule and the oxygen atom in another water molecule is between 1.5 and 2.4 Å, and for a corresponding H–O···O angle of <30°. To calculate the HB density profiles (Figure 18), the position of a HB was taken as mid-distance between the oxygen and hydrogen atom positions in the HB. On both the [110] and [100] surfaces, the low density of HBs near the interface shows that water molecules in the first hydration layer primarily form H-bonds with alumina surface groups, rather than with each other. At distances greater than ∼12 Å, the HB density distributions become uniform, representative of those obtained for bulk water. In the region up to 12 Å, the results show differences that are substrate specific.

For the [110] surface (Panel A), comparison of the HB density peaks to the atomic density profile of OW confirms bonding between the first and second hydration layers, with limited connectivity between the second to third. A pronounced peak at 5.4 Å suggests close association between the third to fourth hydration layers where the population of water accumulates. These results are consistent with the interpretation of the density profiles discussed in Section 3.1.1.

On the [100] surface (Panel B), the HB density profile shows a first peak representing water hydrogen-bonding between the first and second hydration layers. A prominent peak appears within the second hydration layer, where our prior results identified a divergence of water molecule orientations.

The ions cause a reduction in water–water HB densities more pronounced than the changes to water atomic density profiles (SI Figure S8) because of the ions’ ability to perturb the structure of interfacial water. The reduction in water–water HB densities is particularly apparent for barium acetate, in the third to fourth hydration layers of γ-alumina [110], and from the second hydration layer of [100].

## 4 Conclusions

Atomistic molecular dynamics simulations were conducted to investigate interfacial hydration structure, and the effects of ions, at two terminations of gamma-alumina. Atomic density profiles, molecular orientation, 2-D density distributions, and HB density profiles were utilized to assess structural properties at the interfaces, while dynamic properties were quantified in terms of water residence autocorrelation functions.

The results show closer association of water to the [110] surface with clearly defined structural arrangement of interfacial water, resulting in the physical exclusion of ions from the first hydration layer. By comparison, diffuse interfacial water structure at the [100] surface allows closer association of ions, with adsorption of smaller cations (sodium, ammonium) observed on the substrate, and discernible disruption of interfacial water structure in both the first and second hydration layers.

Longer residence times of water in the first hydration layer of γ-alumina [110] are consistent with the closer, tightly held hydration layers at this interface, compared to the [100] surface. While ions associate more closely with the latter, their presence had little influence on interfacial water dynamics, while effects were more pronounced on the [110] surface. The results are interpreted in terms of competition of ion–water and surface–water interactions at the [110] surface.

Interpretation of the differing interfacial behaviors is achieved based on the physical characteristics of the two surfaces. Compared to γ-alumina [110], the [100] surface has a higher density of OH groups (12.9 compared to 10.3 OH/nm^2^) and, in the model implemented, hosts two H_2_O surface groups per unit cell, compared to one for [110]. However, the [110] surface displays more heterogeneity, in terms of contrast between surface features; intimately neighboring areas with and without OH groups, and a degree of roughness (surface “cavities”) at a scale which appears to promote closer association of interfacial water. The [100] surface favors ion-surface interactions, with two surface atom groupings creating localized zones of positive and negative charge balance, attracting anions and cations, respectively. The results presented demonstrate the use of classical MD simulations as an investigative tool to improve characterization and understanding of γ-alumina interfaces, of relevance to wide-ranging practical applications.^7,8,98,99^

## Supporting Information Available

Comparison of RDFs with ab initio data, atomic density profiles (water and ions) perpendicular to γ-alumina surfaces, simulation snapshots of ion adsorption, surface density distributions of ions, overlays of crystallographic surface atoms onto surface density distributions of interfacial water to correlate water arrangement with surface features of [110] and [100] γ-alumina, and crystallographic information for γ-alumina unit cell structure (PDF)
