# Supplementary material for: Hydration Structures on γ-Alumina Surfaces With and Without Electrolytes Probed by Atomistic Molecular Dynamics Simulations
Source: J Phys Chem B. 2022 Nov 2;126(44):9105–22. doi: 10.1021/acs.jpcb.2c06491 (PMC9661474; doi:10.1021/acs.jpcb.2c06491)
Supplement: Supplementary file 1 — jp2c06491_si_001.pdf [file jp2c06491_si_001.pdf]

Supporting Information for:

Hydration Structures on γ-alumina Surfaces With and Without Electrolytes Probed by Atomistic Molecular Dynamics Simulations

Olivera Drecun[a)], Alberto Striolo[a,b,*)], Cecilia Bernardini[c)], Misbah Sarwar[c)]
[a)] *Department of Chemical Engineering, University College London, WC1E 7JE, UK.*
[b)] *School of Chemical, Biological and Materials Engineering, University of Oklahoma, Norman, OK 73019, United States*
[c)] *Johnson Matthey Technology Centre, Sonning Common, Reading RG4 9NH, UK.*
** Corresponding author: astriolo@ou.edu*

Contents

Page #:	
2	**Figure S1.** Effects of system size: water ***ρ***(z) profiles and residence times at γ-alumina [100].
3	**Figure S2.** Radial distribution functions: for surface groups, and surface groups to water; comparison with ab-initio data.
4	**Figure S3.** Atomic density profiles, spanning the complete thickness of the water layer above the γ-alumina [110] and [100] surfaces. Aqueous phase: pure water.
5	**Figure S4.** Water at the [110] γ-alumina interface: simulation snapshots.
6	**Figure S5.** Comparison of water residence times at γ-alumina surfaces, starting from different time origins.
7	**Figure S6a.** Overlay: γ-alumina [110] surface onto **first** interfacial hydration layer water (oxygen and hydrogen) density distributions.
8	**Figure S6b.** Overlay: γ-alumina [110] surface onto **second** interfacial hydration layer water (oxygen and hydrogen) density distributions.
9	**Figure S7a.** Overlay: γ-alumina [100] surface onto **first** interfacial hydration layer water (oxygen and hydrogen) density distributions.
10	**Figure S7b.** Overlay: γ-alumina [100] surface onto **second** interfacial hydration layer water (oxygen and hydrogen) density distributions.
11	**Figure S8.** Complete atomic density profiles, of water and ions, perpendicular to γ-alumina surfaces. Panel 1: [110] γ-alumina. Panel 2: [100] γ-alumina.
12	**Figure S9.** Planar density distributions of water over the γ-alumina [110] surface; influence of salts.
13	**Figure S10.** Surface density distributions of ions, within first and second interfacial hydration layers of γ-alumina [110].
14	**Figure S11.** Surface density distributions of ions, within first and second interfacial hydration layers of γ-alumina [100].
15	**Figure S12.** Barium acetate ion association: [100] γ-alumina interface, second hydration layer, simulation snapshots.
16	**Figure S13.** Planar density distributions of water over the γ-alumina [100] surface; influence of salts.
17	**Figure S14.** Sodium adsorption at the [100] γ-alumina surface: simulation snapshots.
18	**Figure S15.** Ammonium adsorption at the [100] γ-alumina surface: simulation snapshots.
19	**Figure S16.** Nitrate adsorption at the [100] γ-alumina surface: simulation snapshots.
20	**Figure S17.** Chloride interaction with the [100] γ-alumina surface: simulation snapshots.
21	**Figure S18.** Nitrate adsorption at the [110] γ-alumina surface: simulation snapshots.
22	**Section S19.** Crystallographic information file (CIF) contents for the γ-alumina unit cell model of Digne et al.
24	**References**

FIGURE S1.

The smaller of the two systems investigated in the manuscript is the γ-alumina **[100]** surface (surface dimensions: 44.696 * 25.239 * 16.136 Å in x, y and z dimensions, respectively). To determine the extent of any system size effects, a simulation for this surface, with pure water as the aqueous phase, was repeated, but doubling the size of the surface in the y-dimension, yielding a bigger surface size of 44.696 * 50.478 * 16.136 Å. Comparison of results for the 'original' and 'bigger' surface sizes are shown below, in terms of atomic density profiles of water, perpendicular to the surface, and water residence times at the interface.

Density profiles of water, oxygen and hydrogen atoms, are shown in panels **A)** and **B)**, respectively.

Water residence times within the first and second interfacial hydration layers are shown in panels **C)** and **D)**, respectively.

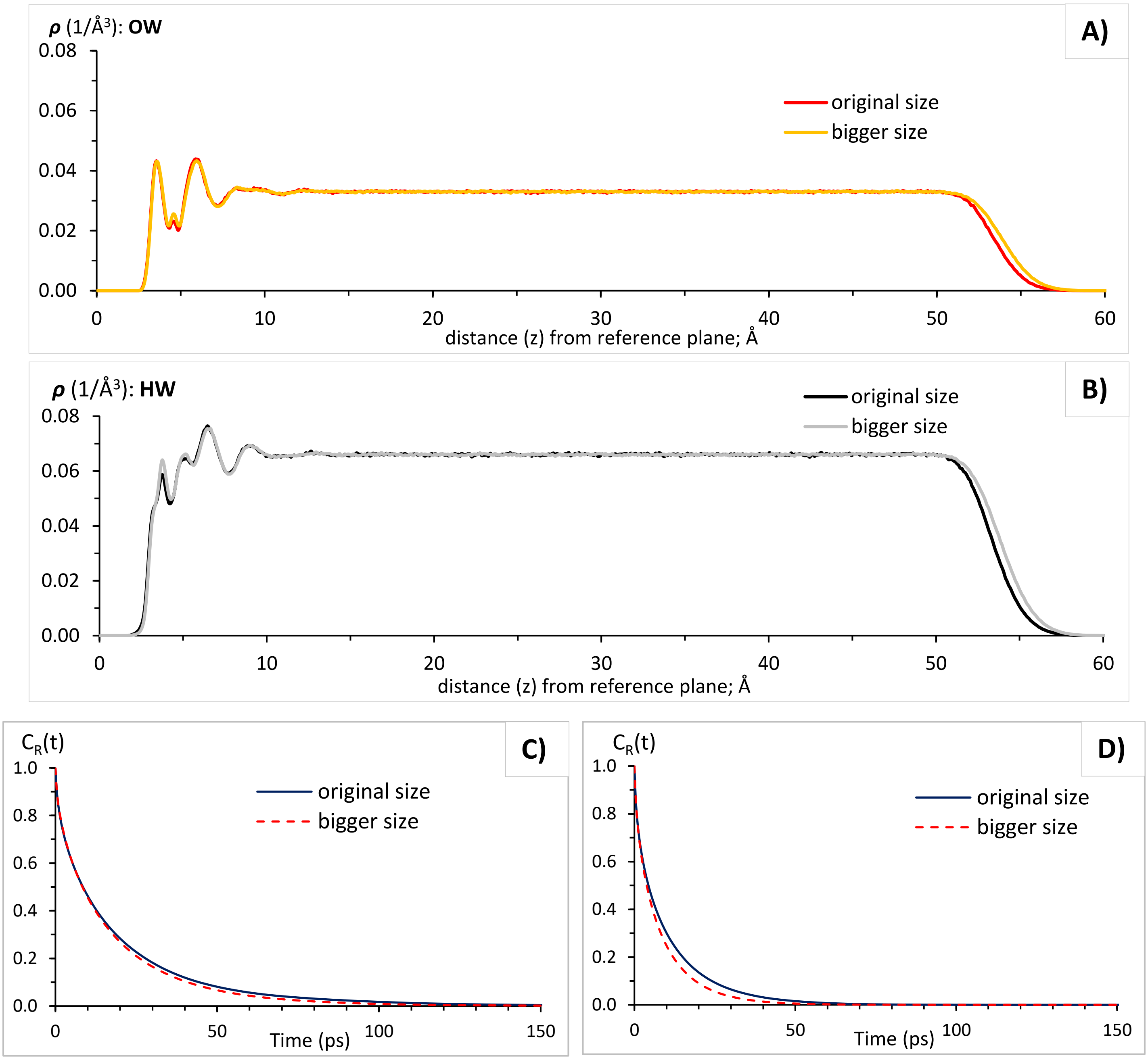

FIGURE S2.

Radial distribution functions (RDFs) obtained for γ-alumina surfaces, compared with selected ab-initio data[1]. Ab-inito data points obtained through DigitizeIt software.

Panels A, B: O (μ_1-OH, μ_3-OH, surface H_2O (μ_1-H2O) groups) vs. H (liquid water), for γ-alumina **[110]** and **[100]** surfaces, respectively. Ab-initio data obtained from Figure 5c, Wakou et al.[1] MD results appear to pick up the main features of the ab-initio data. An exception is for the first peak at 1Å, representing transient protonation of surface OH groups (μ_1-OH and chemisorbed H_2O). At distances of 1.75Å (2Å for MD), bonding occurs between these OH groups and water hydrogens.

Panels C, D: RDFs between all oxygen and hydrogen atoms of **[110]** and **[100]** alumina **surface groups**, respectively; OH pairs **within** μ_1-OH, μ_3-OH and surface H_2O groups (μ_1-H2O). Ab-initio data obtained from Figure S4 (Supplementary Info.) of Wakou et al.[1]

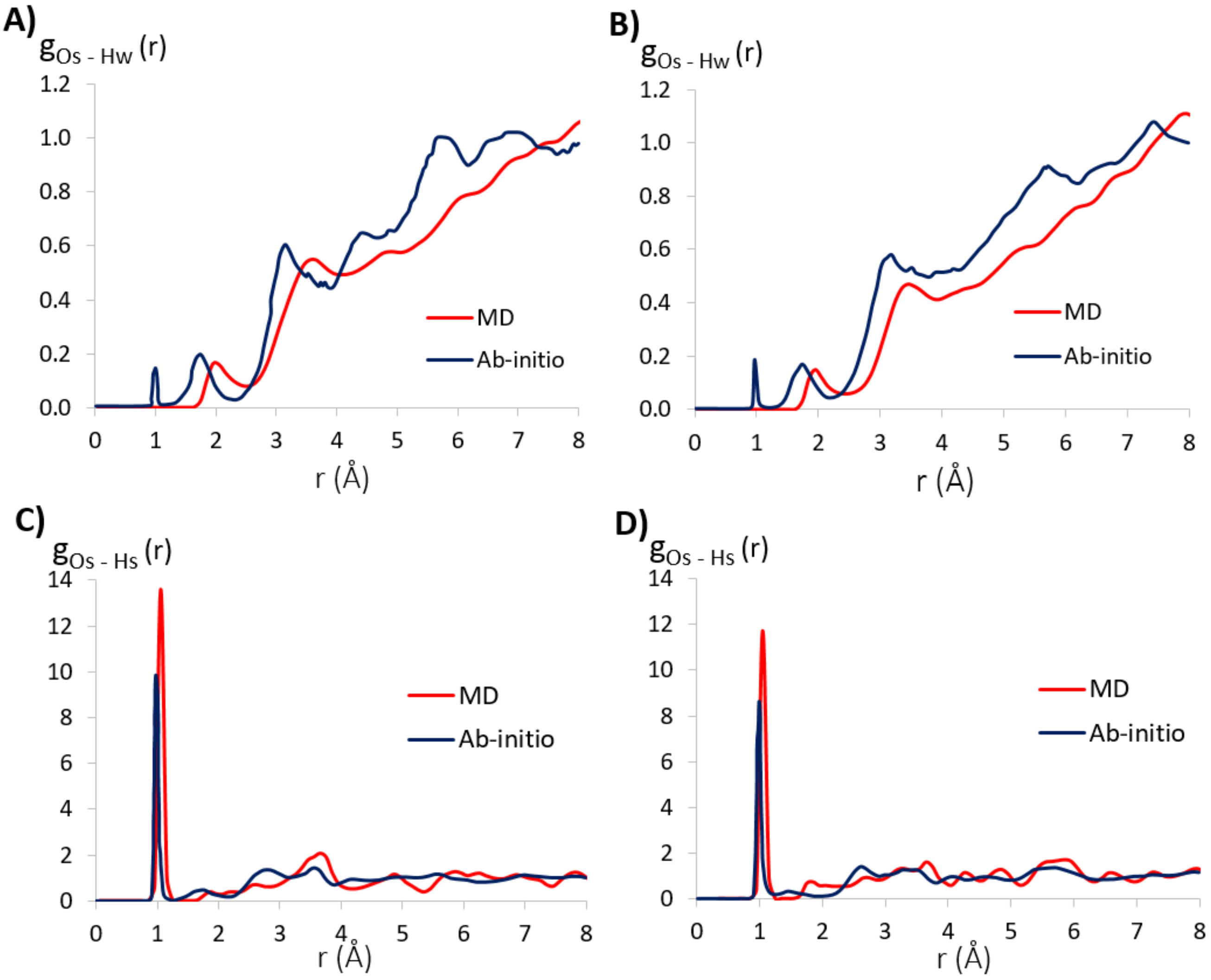

FIGURE S3.

Atomic density profiles of (pure) water perpendicular to γ-alumina surfaces, spanning the complete thickness of the water layer (~50-55 Å); pale red and grey indicate water oxygen and hydrogen atoms, respectively. Profiles over the thickness considered in the main text (15 Å) are shown alongside (in red and black, for water oxygen and hydrogen, respectively).

Panel A: atomic density profiles, [110] surface. **Panel B:** atomic density profiles, [100] surface. Simulation box dimensions and the amount of water incorporated is sufficient to ensure that 'bulk-like' water conditions are established in the middle of the layer, away from the influence of the two interfacial regions; solid-liquid and liquid-vacuum, respectively.

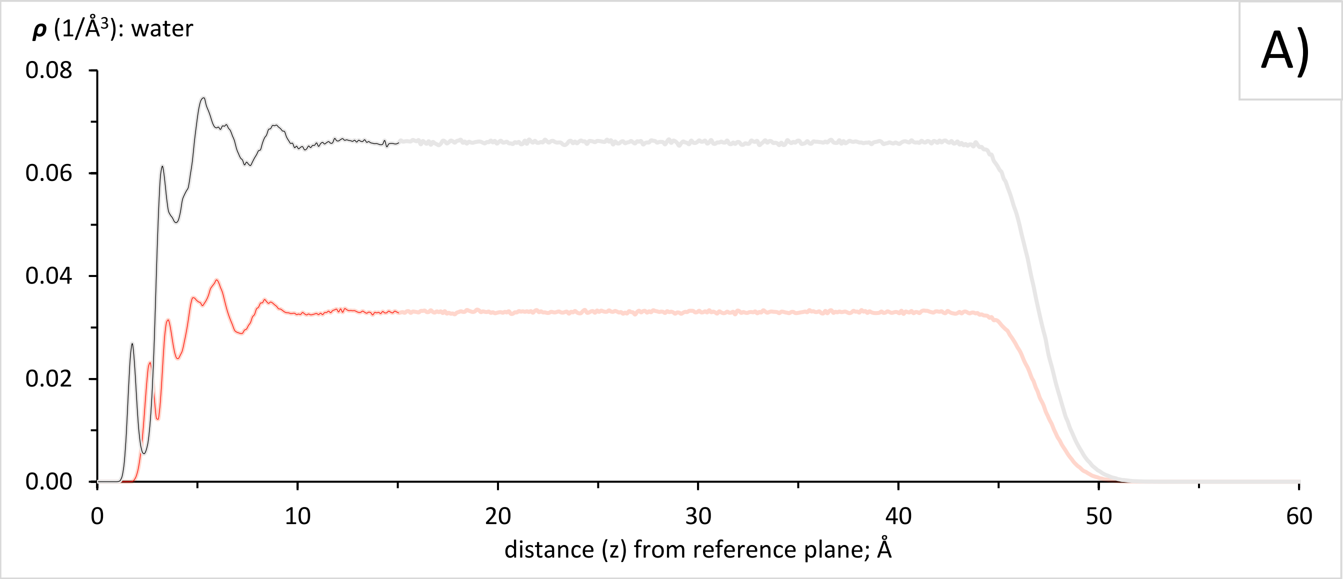

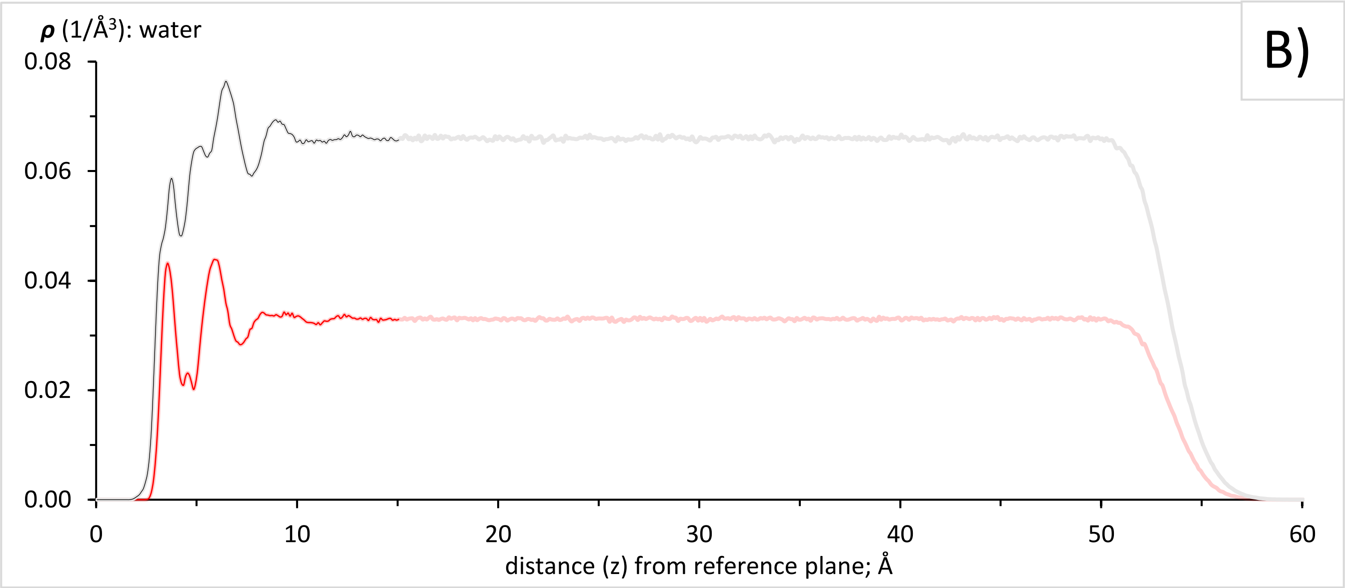

FIGURE S4.

Simulation snapshots from the γ-alumina [110] surface, showing water within the first and second hydration layers. Selected surface atoms from the aerial view are labelled for position reference. Dynamic bonds are shown in the snapshots, colour-coded to illustrate the transition from the first to the second hydration layer. Green; interaction between the surface and water molecules in the first hydration layer. Pale yellow; interaction between water molecules in the first and second hydration layers (i.e., the transition between layers). Cyan; interaction of water molecules in the second hydration layer with alumina surface groups. Lengths of the dynamic bonds shown range from 1.7 to 1.9 Å.

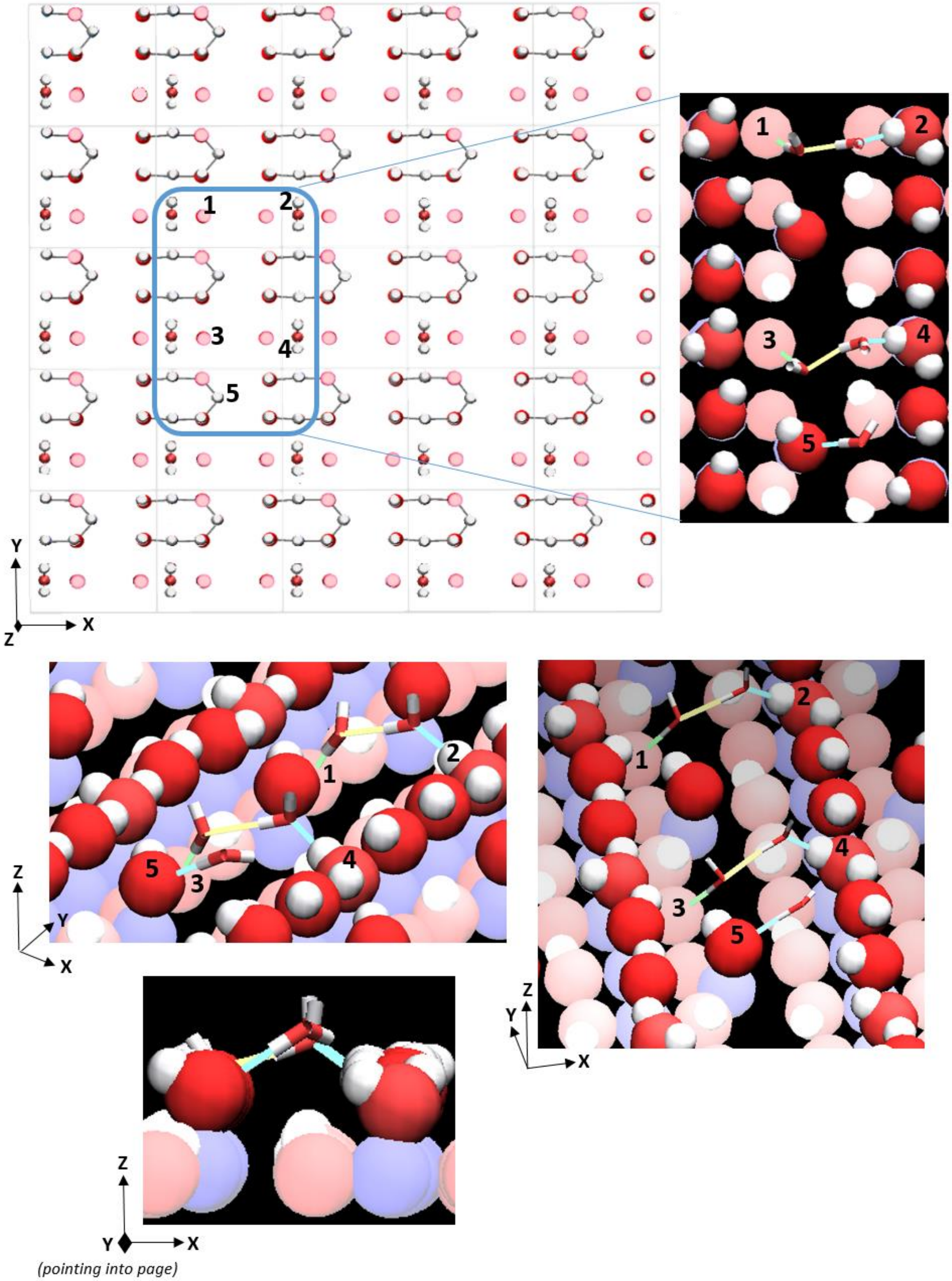

FIGURE S5.

To provide an indication of uncertainty for the computed average water residence times, the analysis was repeated, starting from different time origins within the simulation trajectory output. The full simulation trajectory utilised for the present work comprises 10,000 frames (each frame = 400 fs duration). Average residence times presented in the main text were computed between frames 5000-10,000. Results below show the same analysis, starting from frame zero (to 5000), and frames 2500-7500.

Panels A), B): γ-alumina **[110]**, first and second interfacial hydration layers, respectively.

Panels C), D): γ-alumina **[100]**, first and second interfacial hydration layers, respectively.

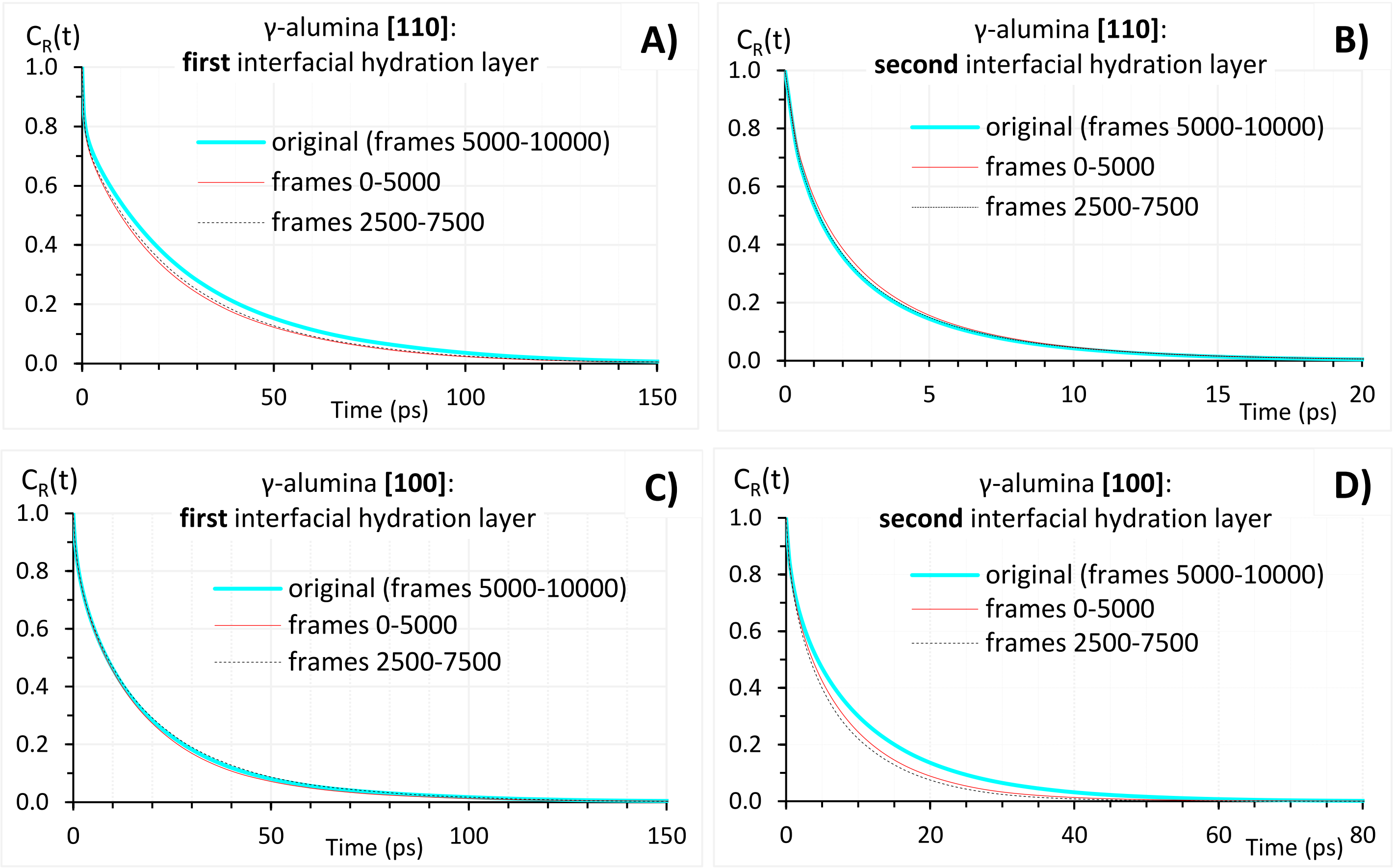

FIGURE S6a.

The [110] γ-alumina surface, superimposed onto surface density distribution graphs of interfacial water (taken from main text Fig. 5, row A). For clarity, only the surface atoms of [110] γ-alumina are shown. Diagrams A) and B) – **first** interfacial hydration layer; water oxygen and hydrogen density distributions, respectively. Unit cells (white borderlines) are shown for clarity. White = hydrogen, red (where visible) = oxygen atoms part of an OH group, pale pink = free surface oxygen atoms. Scale bar is applicable to both graphs.

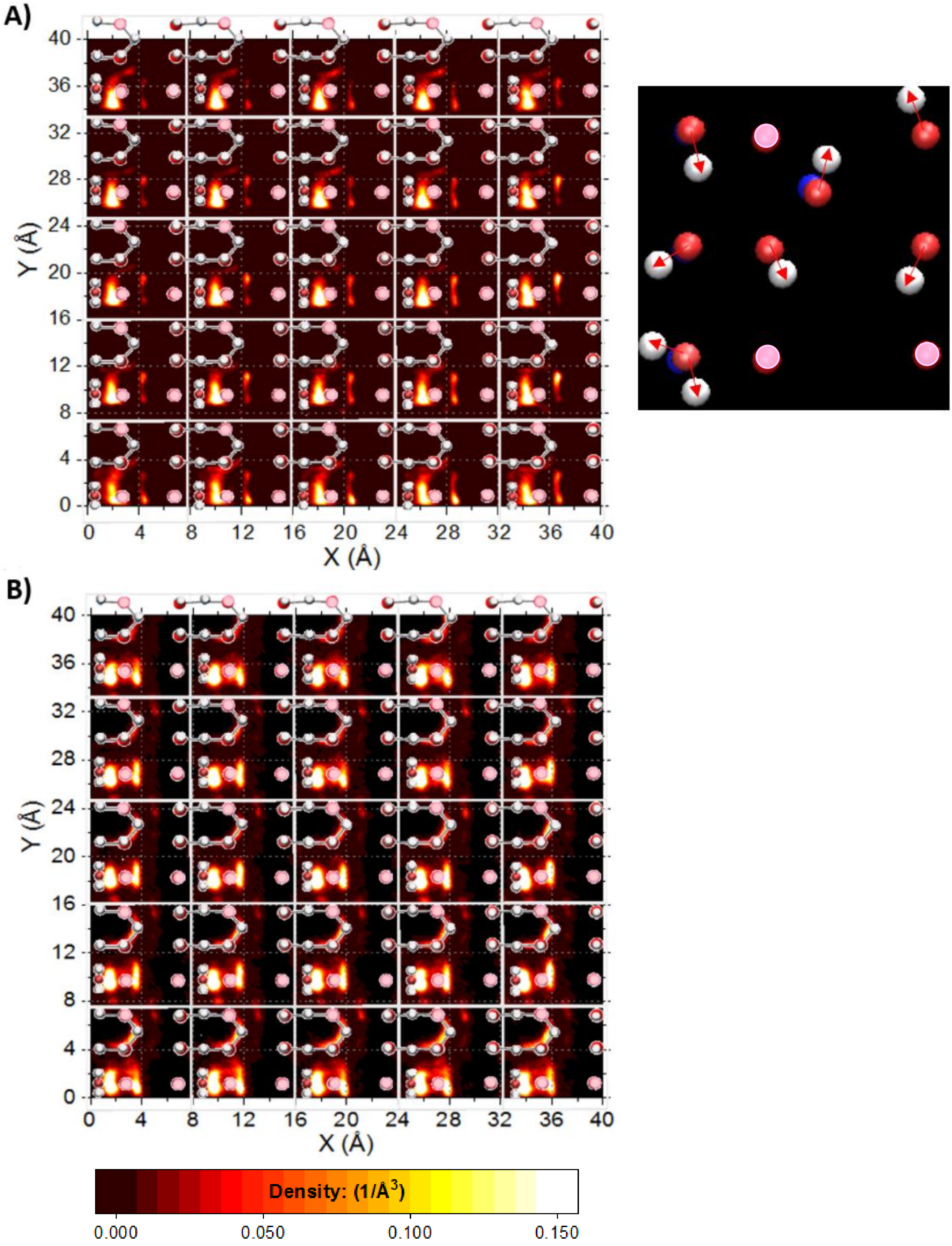

FIGURE S6b.

The [110] γ-alumina surface, superimposed onto surface density distribution graphs of interfacial water (taken from main text Fig. 5, row A). For clarity, only the surface atoms of [110] γ-alumina are shown. Diagrams A) and B) - **second** interfacial hydration layer; water oxygen and hydrogen density distributions, respectively. Unit cells (white borderlines) are shown for clarity. White = hydrogen, red (where visible) = oxygen atoms part of an OH group, pale pink = free surface oxygen atoms. Scale bar is applicable to both graphs.

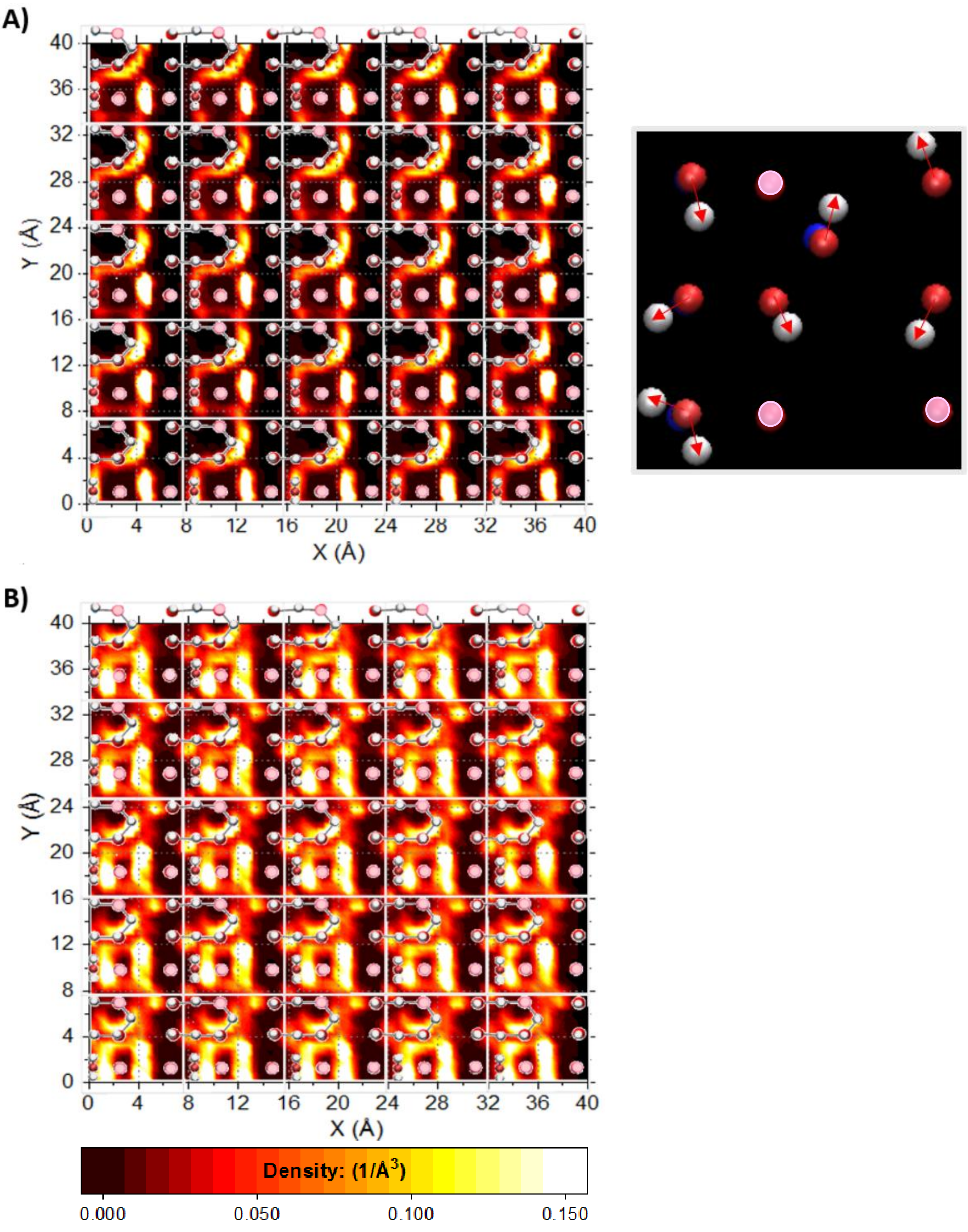

FIGURE S7a.

The [100] γ-alumina surface, superimposed onto surface density distribution graphs of interfacial water (taken from main text Fig. 6, row A). For clarity, only the surface atoms of [100] γ-alumina are shown. Diagrams A) and B) – **first** interfacial hydration layer; water oxygen and hydrogen density distributions, respectively. Unit cells (white borderlines) are shown for clarity. OH groups are shown in initial configuration (bond vectors aligned in the direction normal to the surface). See simulation snapshot, showing a single unit cell surface, for representative orientations (indicated with red arrows). White = hydrogen, red (where visible) = oxygen atoms part of an OH group, pale pink = free surface oxygen atoms. Scale bar is applicable to both graphs.

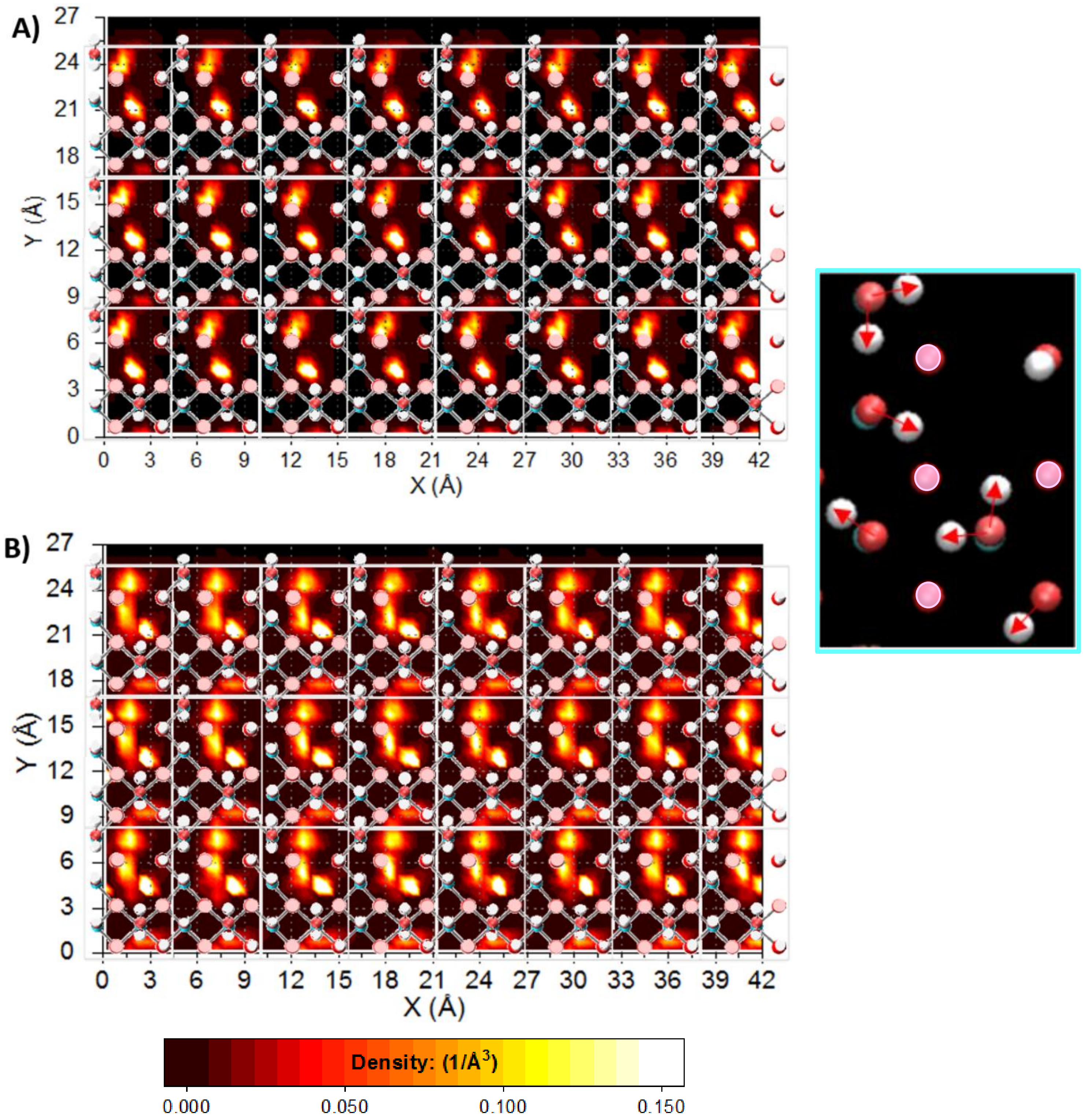

FIGURE S7b.

The [100] γ-alumina surface, superimposed onto surface density distribution graphs of interfacial water (taken from main text Fig. 6, row A). For clarity, only the surface atoms of [100] γ-alumina are shown. Diagrams A) and B) – **second** interfacial hydration layer; water oxygen and hydrogen density distributions, respectively. Unit cells (white borderlines) are shown for clarity. OH groups are shown in initial configuration (bond vectors aligned in the direction normal to the surface). See simulation snapshot, showing a single unit cell surface, for representative orientations (indicated with red arrows). White = hydrogen, red (where visible) = oxygen atoms part of an OH group, pale pink = free surface oxygen atoms. Scale bar is applicable to both graphs.

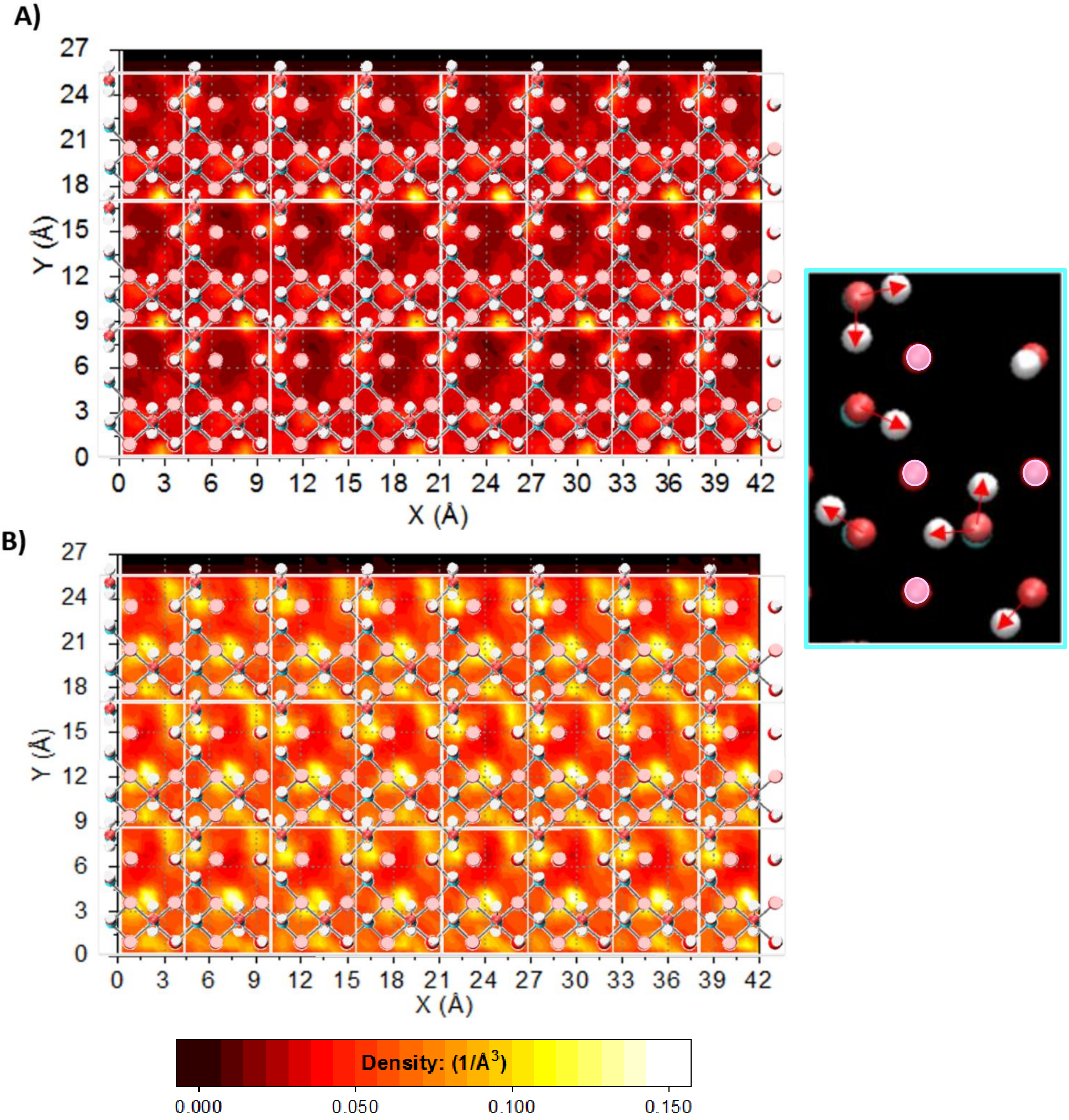

FIGURE S8.

Atomic density profiles, perpendicular to γ-alumina surfaces.

Panel 1: [110] γ-alumina. **Panel 2:** [100] γ-alumina. **Rows A, B:** water (oxygen), water (hydrogen) density profiles, respectively, for all systems, to facilitate comparison of any salt-specific effects. **Rows C to F:** sodium chloride (1 molar, aq.), ammonium acetate (1 molar, aq.), barium nitrate (0.3 molar, aq.), barium acetate (1 molar, aq.).

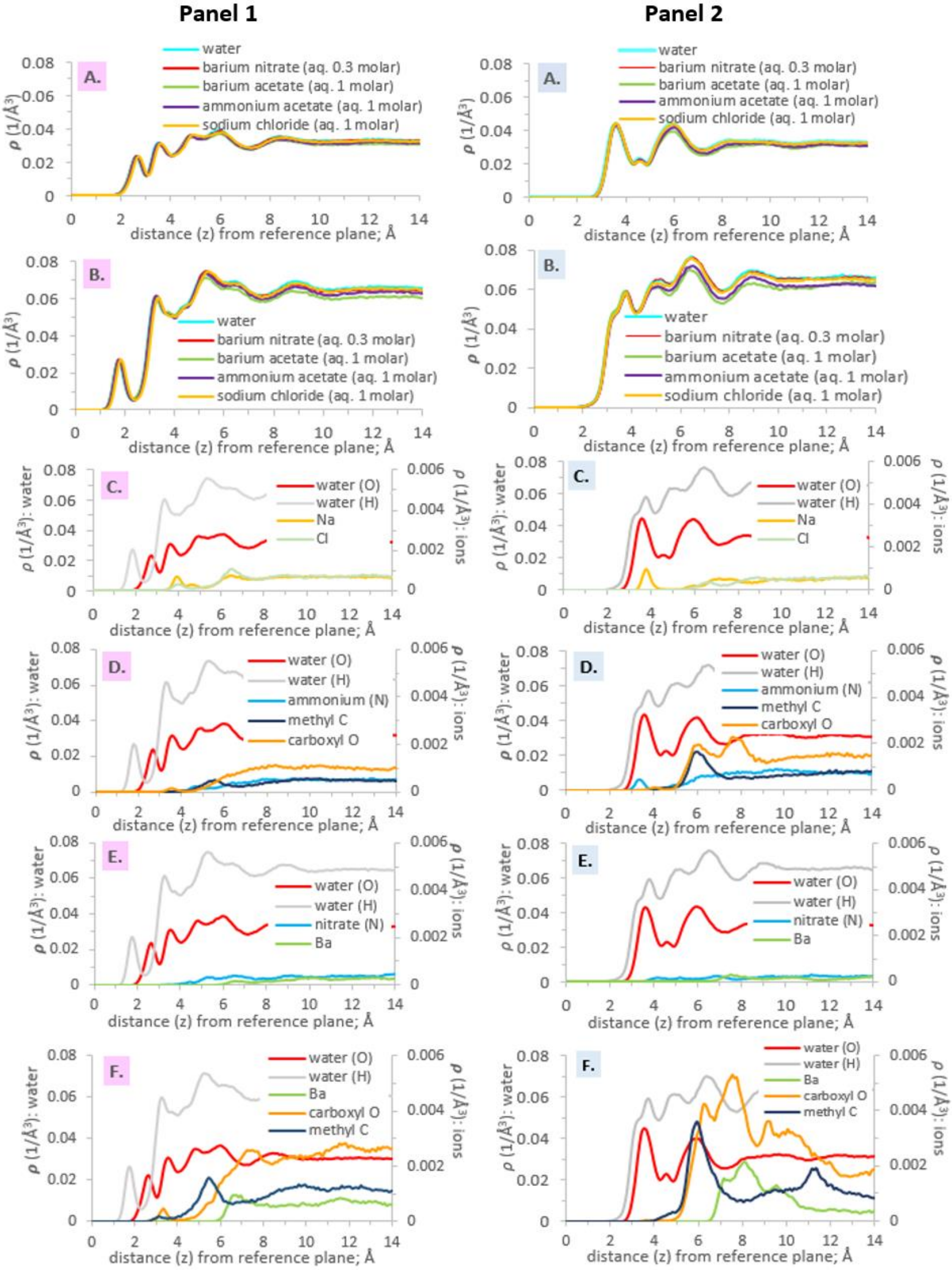

Figure S9.

Planar density distributions of water over the γ-alumina [110] surface. **Columns 1 and 2**: first hydration layer, water oxygen and hydrogen (OW and HW), respectively. **Columns 3 and 4**: second hydration layer, water oxygen and hydrogen (OW and HW), respectively. Rows A), B), C), D) and E) are for pure water, 1M aqueous solution of sodium chloride, 1M aqueous solution of ammonium acetate, 0.3M aqueous solution of barium nitrate, and 1M aqueous solution of barium acetate, respectively. The scale bar is applicable to all graphs in the figure.

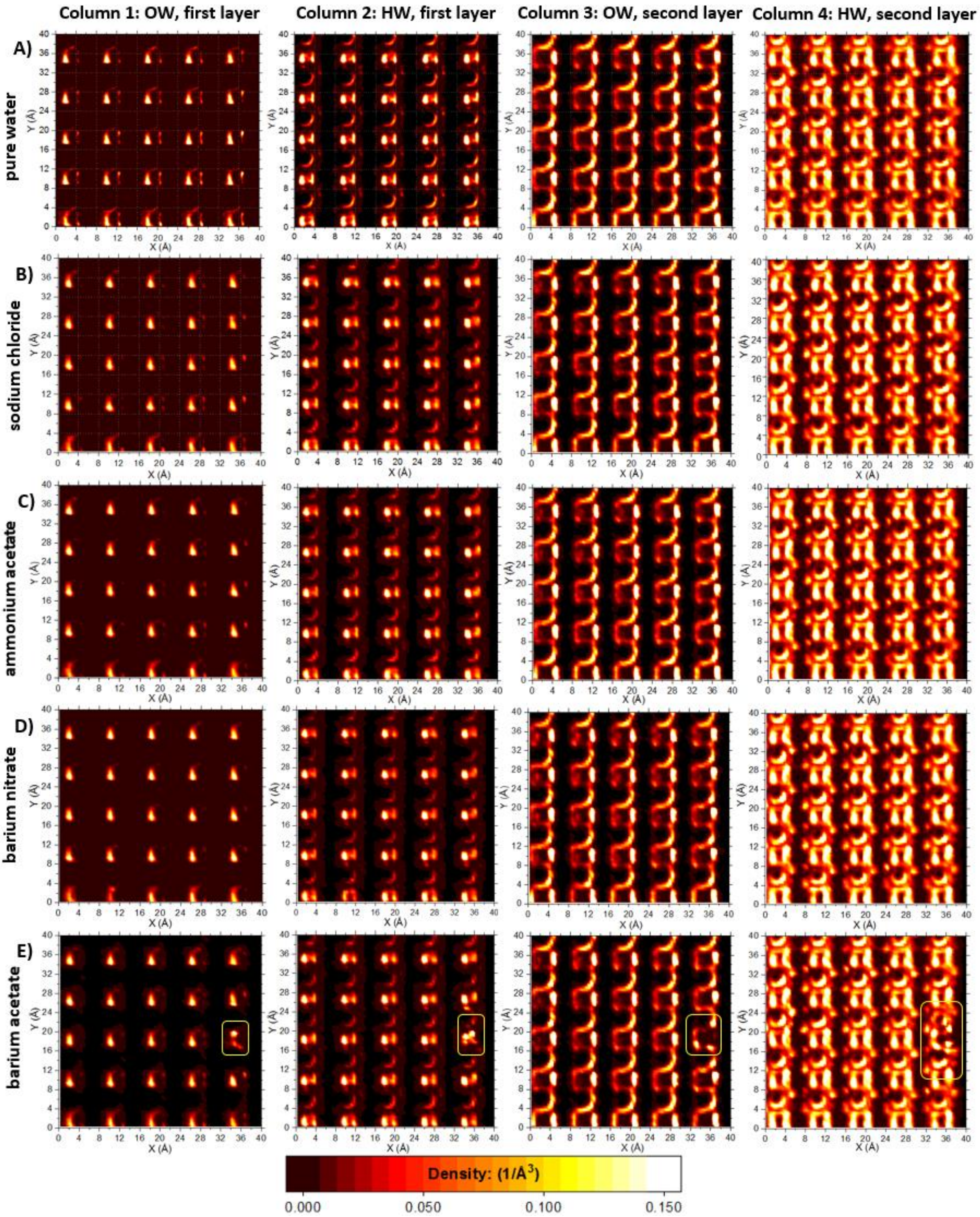

FIGURE S10.

Surface density distributions of ions, within first and second interfacial hydration layers of γ-alumina **[110]**. Labels, 1) and 2), are used to indicate ion density distributions within the first and second hydration layers, respectively. Scale-bar density units: 1/Å^3. Panels A, B, C and D correspond to aqueous systems of 1 molar sodium chloride, ammonium acetate, barium acetate and 0.3 molar barium nitrate, respectively. Feature enlargements are outlined in white; placed in blank areas.

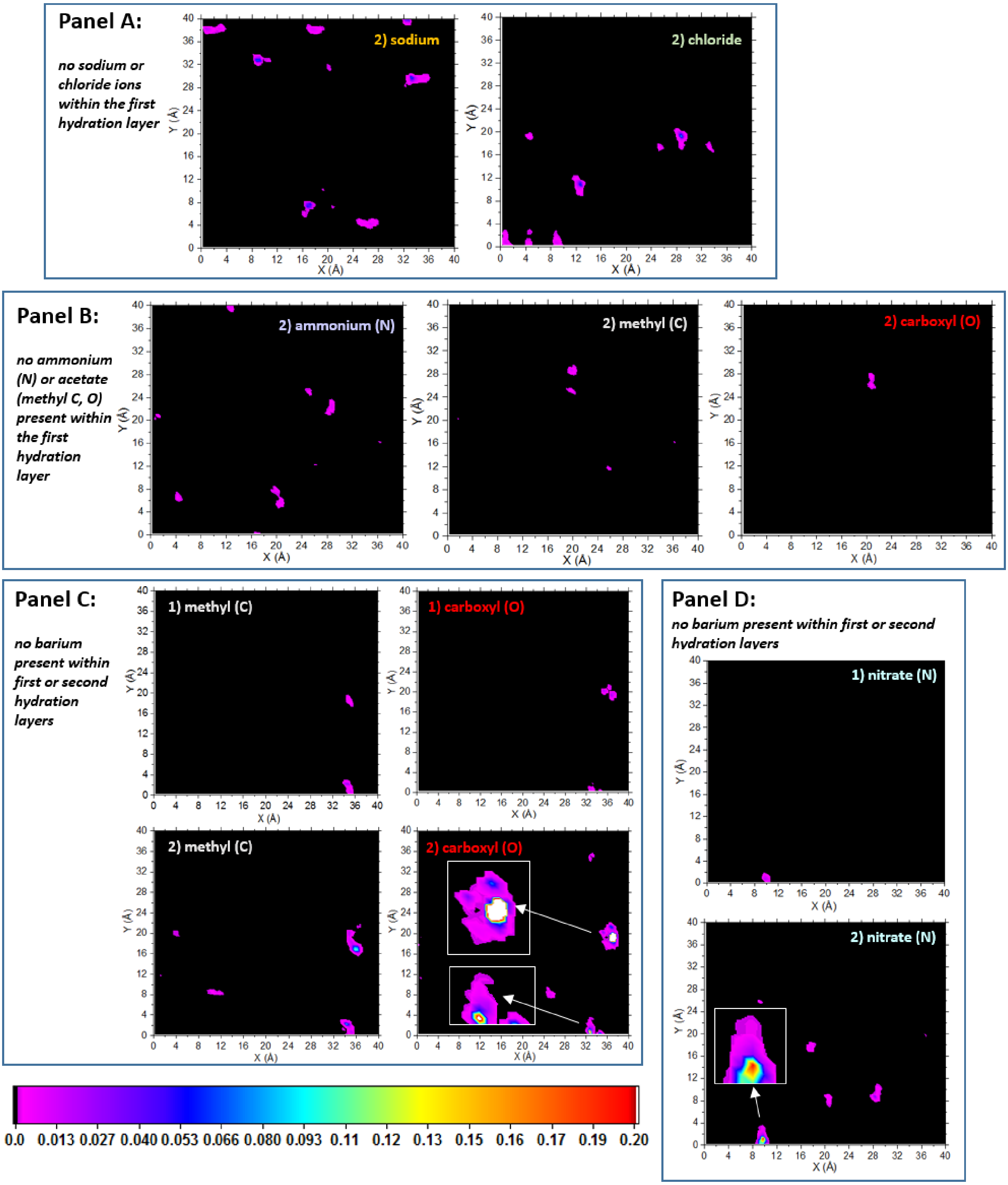

FIGURE S11.

Surface density distributions of ions, within first and second interfacial hydration layers of γ-alumina **[100]**. Labels, 1) and 2), refer to ion density distributions within first and second hydration layers, respectively. Density units: 1/Å^3. Panel A: sodium chloride (1 molar). Panels B, C: ammonium acetate (1 molar), barium nitrate (0.3 molar). Panel D: barium acetate (1 molar). Feature enlargements are outlined in white; placed in blank areas.

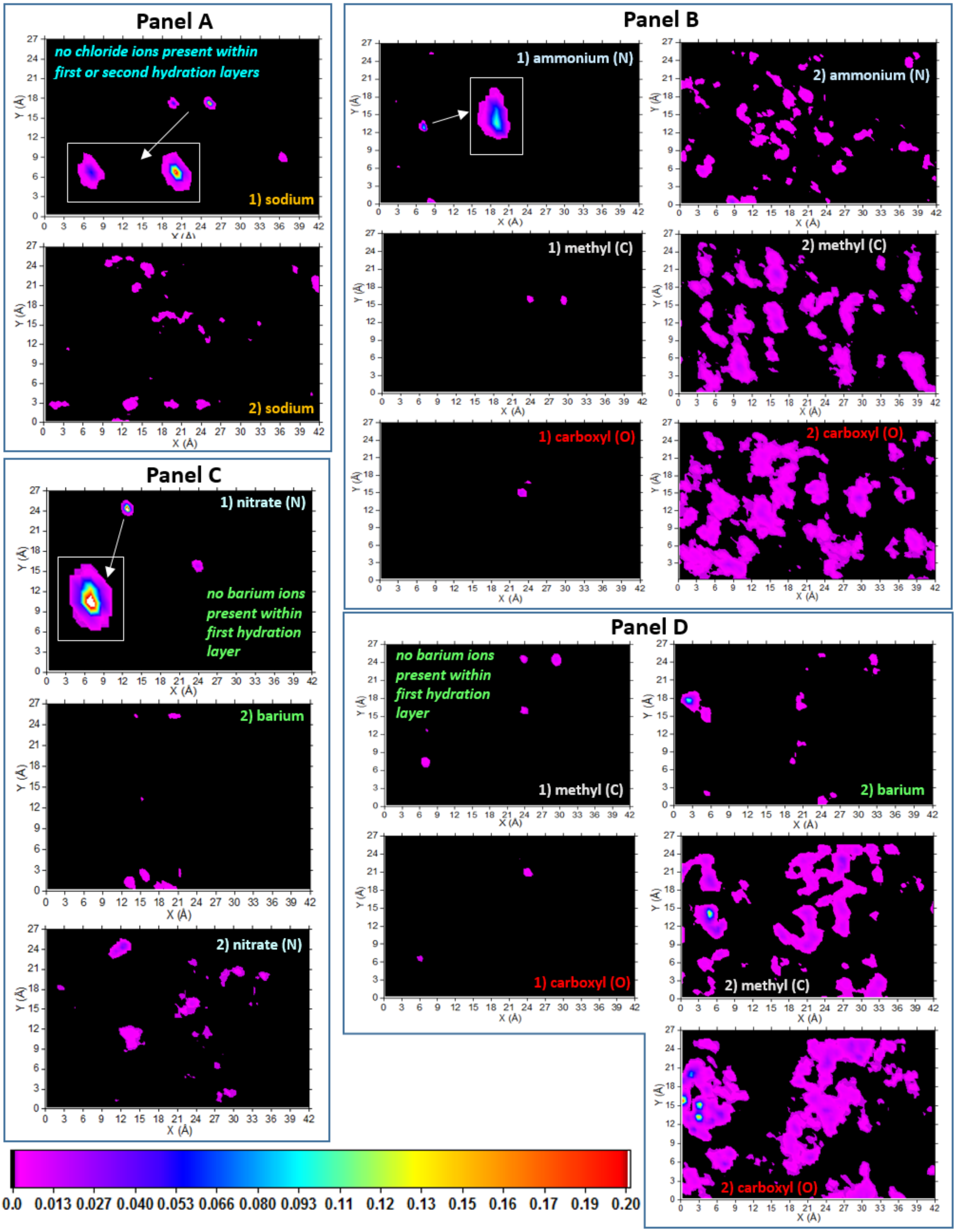

FIGURE S12.

[100] γ-alumina surface, aqueous phase: barium acetate (1 molar).

Panel A) aerial view (initial configuration with unit cells shown, for clarity). Selected atoms are labelled for position reference. A region of complex ion association, as seen during the simulation, is superimposed; an enlargement, minus surface atoms, is provided for clarity. This region of ion accumulation, in the second hydration layer, is visible in **Fig. S10 (Panel D)** and appears to be responsible for interfacial water displacement within this layer, as seen in **Figure 10, row E (main text)**.

Panel B) simulation snapshots. Atoms of the reference plane are shown in faded colours, to aid visualisation. The dynamic bonds depicted between bariums and acetate carboxyl oxygens are of 2.8 Å length. This equates to the distance to the first peak of the radial distribution function for Ba - carboxyl oxygens, in aqueous solution (see Drecun et al., 2021; supplementary information, Figure S2) [2].

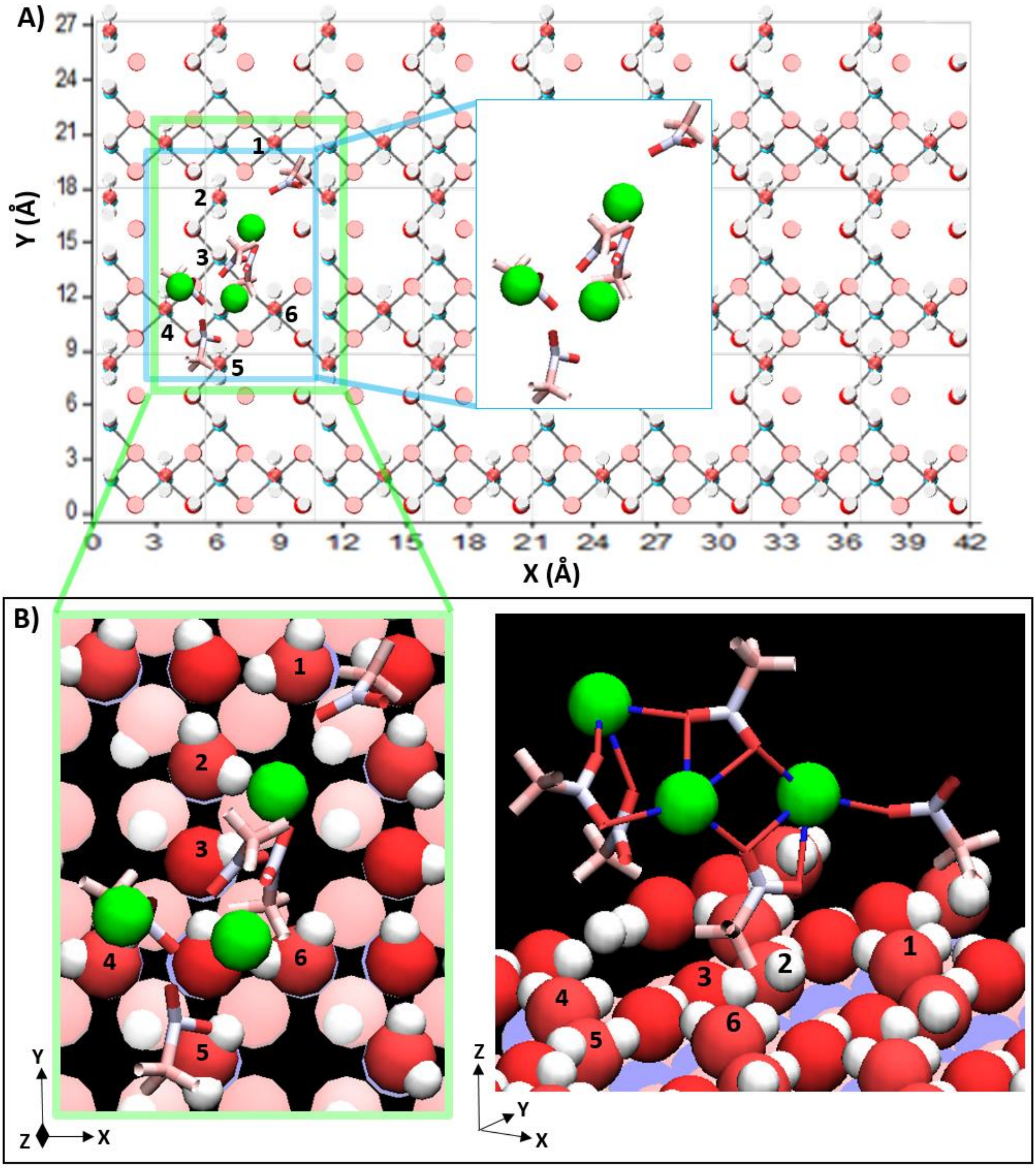

Figure S13.

Planar density distributions of water over the γ-alumina [100] surface. **Columns 1 and 2**: first hydration layer, water oxygen and hydrogen (OW and HW), respectively. **Columns 3 and 4**: second hydration layer, water oxygen and hydrogen (OW and HW), respectively. Rows A), B), C), D) and E) are for pure water, 1M aqueous solution of sodium chloride, 1M aqueous solution of ammonium acetate, 0.3M aqueous solution of barium nitrate, and 1M aqueous solution of barium acetate, respectively. The scale bar is applicable to all graphs in the figure.

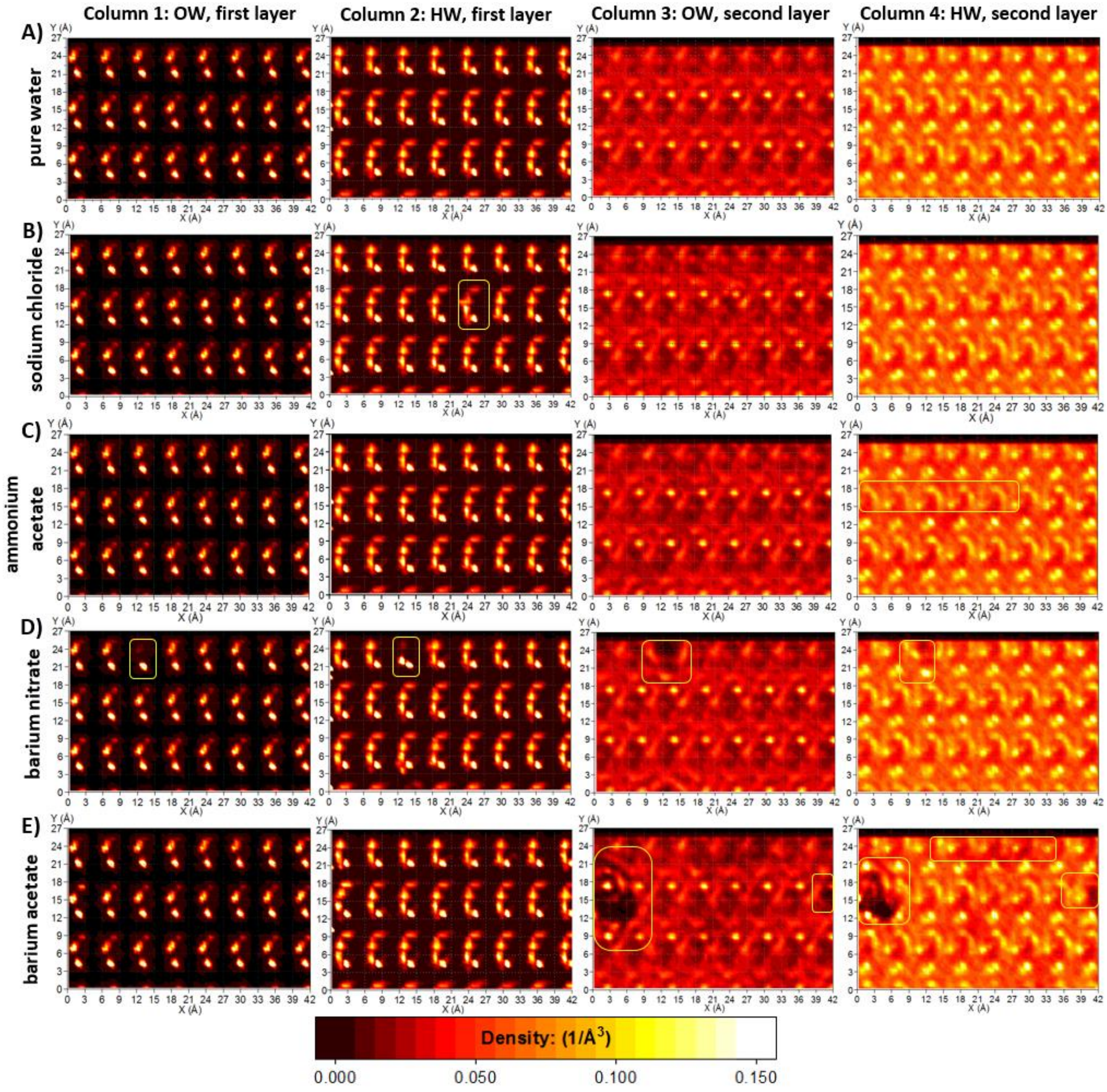

FIGURE S14.

[100] γ-alumina surface, aqueous phase: sodium chloride (1 molar).

Panel A) aerial view of surface (initial configuration with unit cells shown, for clarity). An example adsorption site of Na+ (yellow, label 4), as seen during the simulation, is superimposed. Nearest neighbours of the sodium ion at this site - oxygen atoms, from two Al_4O_1H surface groups, and an $Al_5O_1H_2$ group - are labelled 1), 3) and 2), respectively.

Panel B) simulation snapshots showing sodium ion interaction at the adsorption site and orientation of alumina surface groups, with a water molecule (stick representation) appearing in the first hydration layer that appears to 'stabilise' the configuration (label 5). Atoms of the plane below (reference plane) are shown in faded colours, to aid visualisation. Atom coordinates from the snapshots (x, y, z):

1) 5.66, 4.58, 19.62 2) 8.38, 1.86, 19.55 3) 11.14, 4.58, 19.65 4) 8.19, 4.17, 19.78 = sodium ion

5) 8.70, 6.48, 20.25 = water (oxygen atom)

In Panel A, another observed adsorption site of sodium is shown (label 6), where the same configuration, with a 'stabilising' water molecule, is observed.

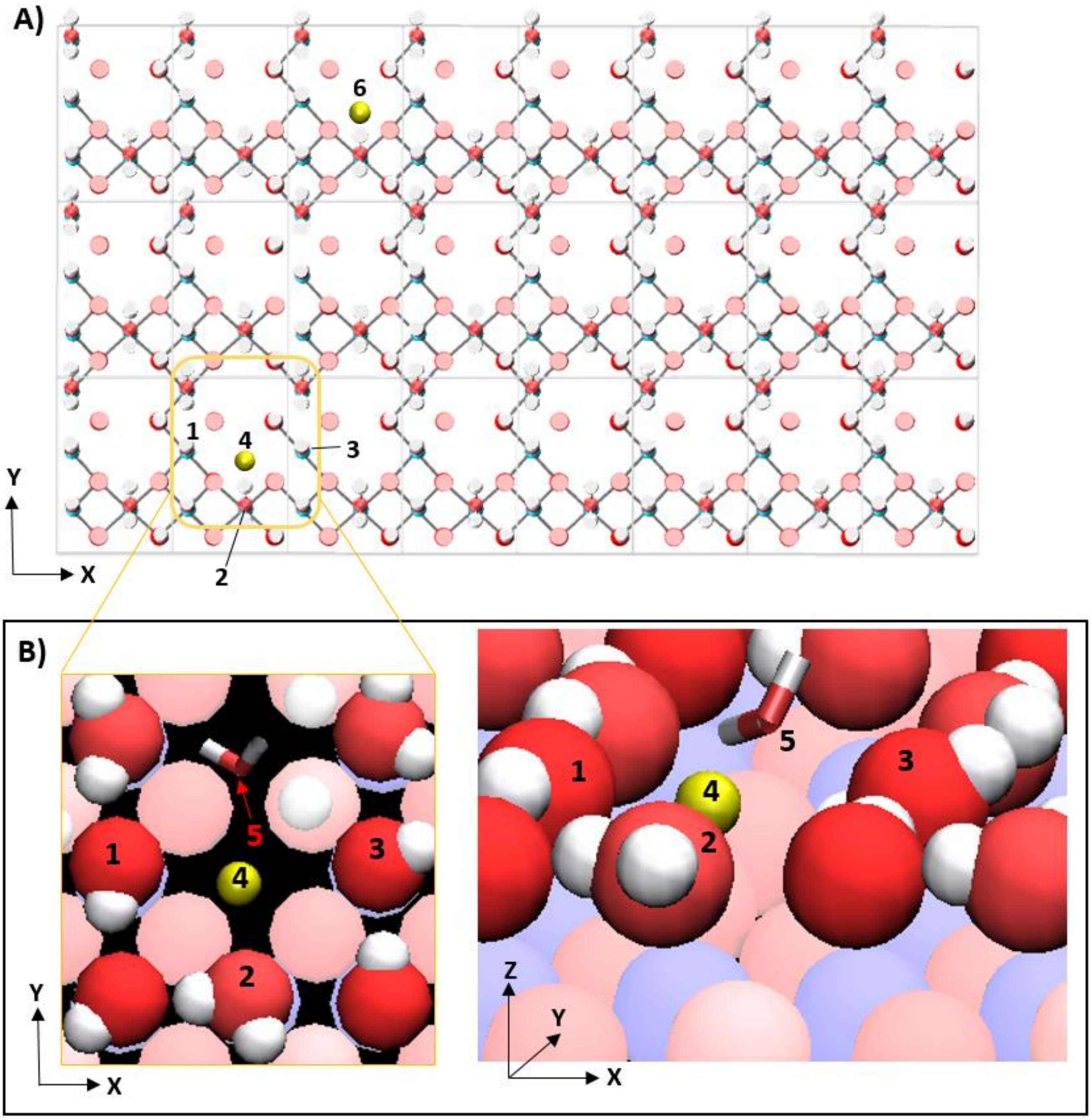

FIGURE S15.

[100] γ-alumina surface, aqueous phase: ammonium acetate (1 molar).

Panel A) aerial view of surface (initial configuration with unit cells shown, for clarity). An example adsorption site of ammonium (label 6), as seen during the simulation, is superimposed. Nearest neighbours of the ammonium ion at this site - oxygen atoms, from four Al_4O_1H surface groups, and an $Al_5O_1H_2$ group - are labelled 1-4, and 5, respectively.

Panel B) simulation snapshots showing ammonium ion interaction at the adsorption site and orientation of alumina surface groups, with a water molecule (stick representation) appearing in the first hydration layer that appears to 'stabilise' the configuration (label 7). Atoms of the reference plane are shown in faded colours, to aid visualisation. Atom coordinates from the snapshots (x, y, z):

1) 5.75, 13.08, 19.59 **2)** 11.22, 13.13, 19.63 **3)** 5.70, 10.26, 19.59 **4)** 11.26, 10.25, 19.73

5) 8.30, 10.24, 19.44 **6)** 8.62, 12.65, 20.80 = ammonium ion (nitrogen) **7)** 8.34, 15.53, 20.52 = water (oxygen atom)

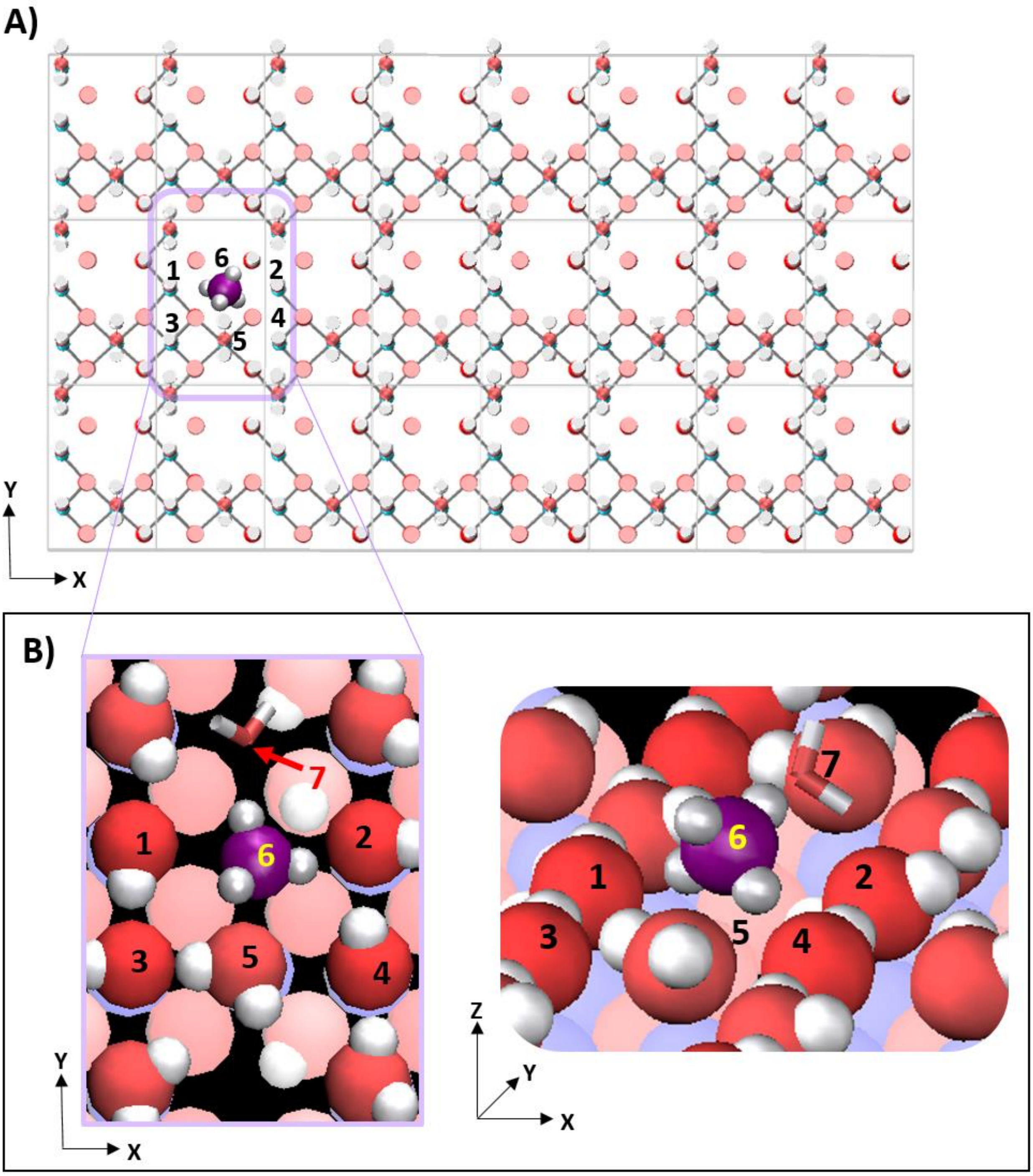

FIGURE S16.

[100] γ-alumina surface, aqueous phase: barium nitrate (0.3 molar).

Panel A) aerial view of surface (initial configuration with unit cells shown, for clarity). Two example nitrate ion adsorption sites (labels 1 and 5), as seen during the simulation, are superimposed. Each nitrate ion appears to interact with three $Al_5O_1H_2$ surface groups, labelled 2-4 and 6-8, respectively.

Panel B) simulation snapshots showing nitrate ion interaction at adsorption sites, and orientation of alumina surface groups. Atoms of the reference plane are shown in faded colours, to aid visualisation.

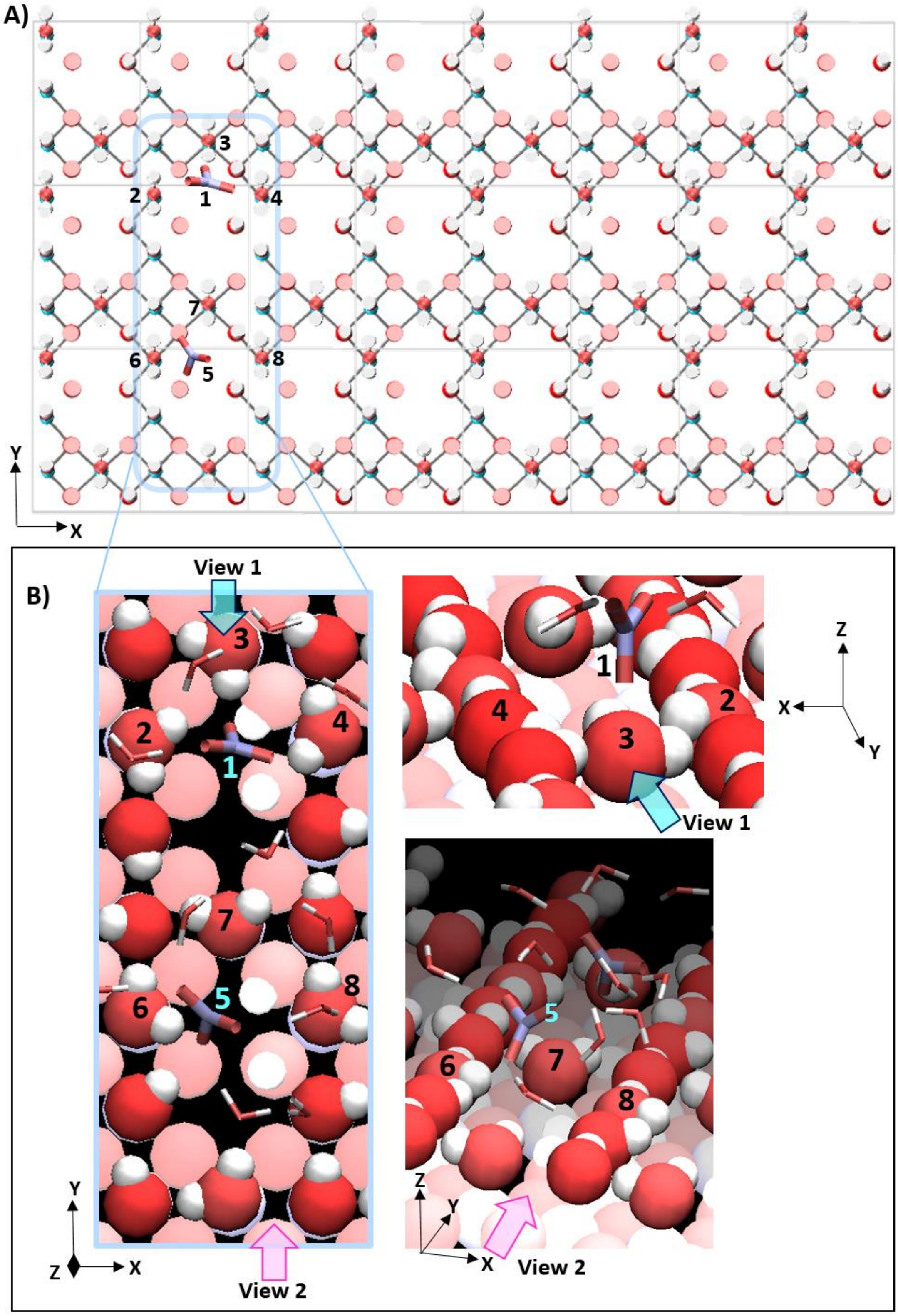

FIGURE S17.

[100] γ-alumina surface, aqueous phase: sodium chloride (1 molar).

Panel A) aerial view of surface (initial configuration with unit cells shown, for clarity). An example adsorption site of Cl- (green, label 2), as seen during the simulation, is superimposed. Nearest neighbour atoms of the chloride ion at this site - hydrogens from two $Al_5O_1H_2$ surface groups - are labelled 1) and 3) respectively.

Panel B) simulation snapshot showing chloride ion interaction at the adsorption site, and orientation of alumina surface groups (water molecules not shown for clarity). Atoms of the plane below (reference plane) are shown in faded colours, to aid visualisation. Atom coordinates from the snapshot (x, y, z):

1) 12.20, 24.24, 19.77 **2)** 14.03, 24.03, 20.94 = chloride ion **3)** 15.70, 24.32, 19.86

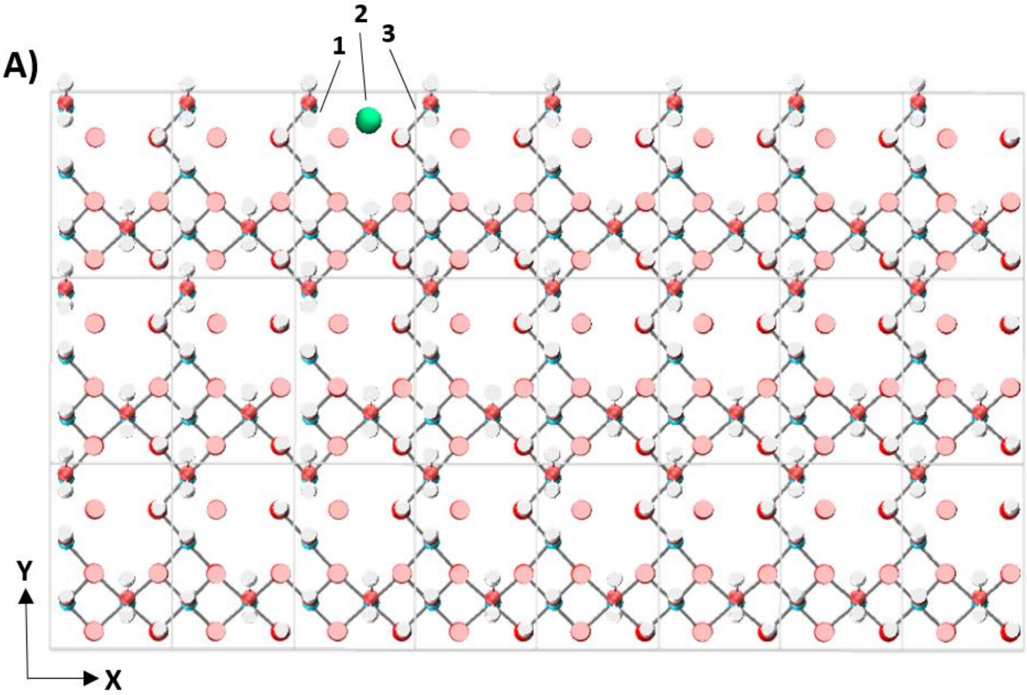

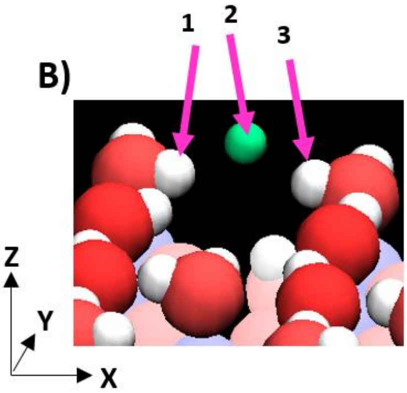

FIGURE S18.

[110] γ-alumina surface, aqueous phase: barium nitrate (0.3 molar).

Panel A) aerial view (initial configuration with unit cells shown, for clarity). Selected atoms of the surface are labelled for position reference. The outlined area (pink) corresponds to a zone of nitrate ion – interfacial water interaction observed during the simulation; shown in the enlargement.

Panel B) simulation snapshot, from different angles. Atoms of the 'reference plane' are shown in faded colours, to aid visualisation. Dynamic bonds are shown in the snapshots (cyan), to illustrate surface – water – ion interaction. Lengths of the dynamic bonds depicted range from 1.7 to 1.9 Å. Water, nitrate ion are depicted with stick representation. During visits to the interface, the nitrate ion appears near the preferred adsorption site of water in the first hydration layer (compare with snapshots in **Fig. S8** for aqueous phase: pure water).

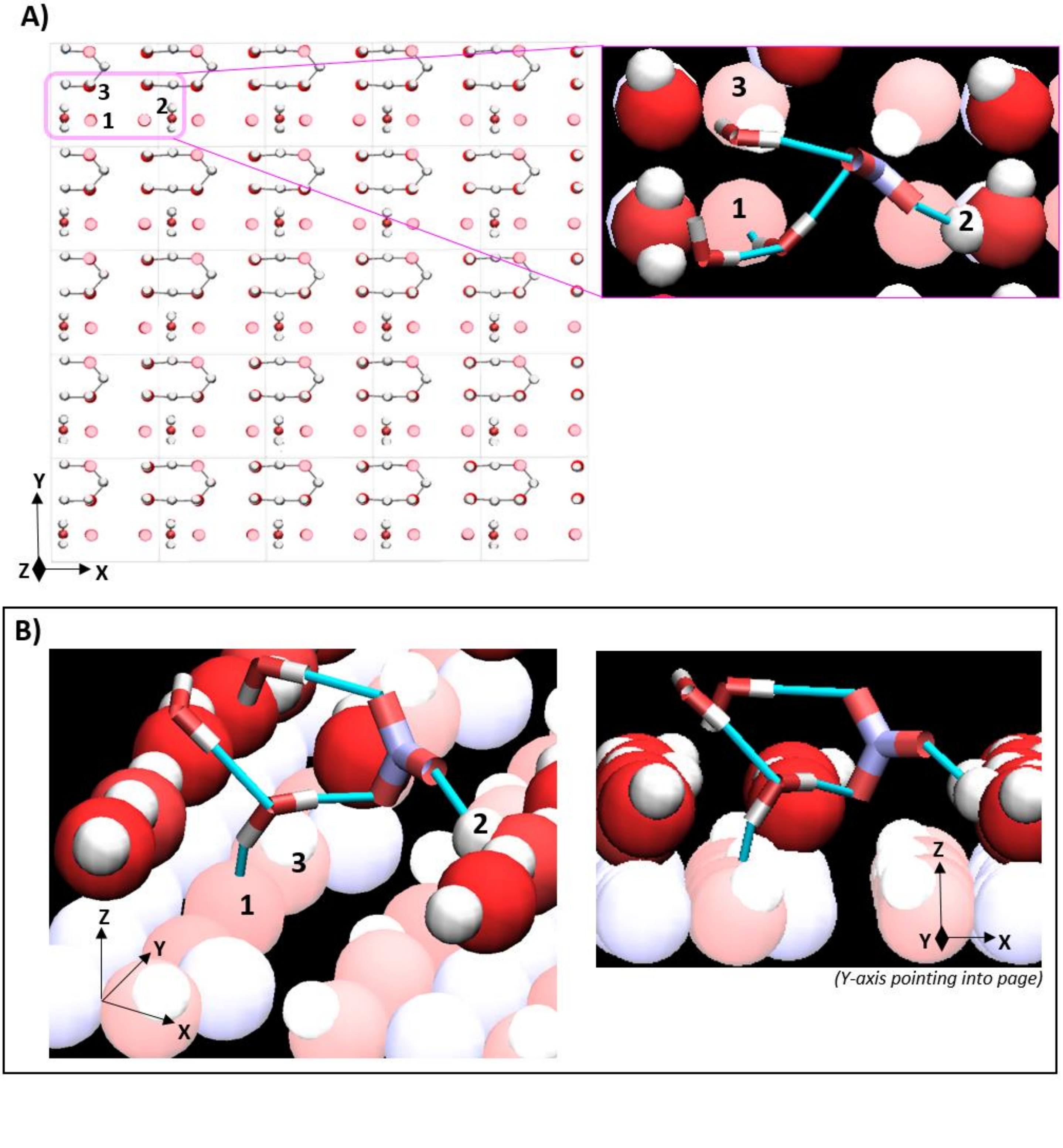

SECTION S19.

Crystallographic information file (CIF) contents for the γ-alumina unit cell model of Digne et al. Template by A. Herráez, modified by J. Gutow. The '.CIF' file contents are provided below (copy and paste into a text file with .CIF extension). Template with further details available/sourced from:
http://www.unm.edu/~ejpete/jmol/gamma_digne_etal/gamma_digne_etal.html

```
data_GAMMA_publ


# 8. Phase information from GSAS

_pd_phase_name                         gamma
_cell_length_a                         5.587
_cell_length_b                         8.413
_cell_length_c                         8.068
_cell_angle_alpha                      90.0
_cell_angle_beta                       90.59
_cell_angle_gamma                      90.0
_cell_volume                           379.20355
_symmetry_cell_setting                 monoclinic
_symmetry_space_group_name_H-M         "P 21/m"
loop_ _symmetry_equiv_pos_site_id _symmetry_equiv_pos_as_xyz
       1 +x,+y,+z
       2 -x,+y+1/2,-z
      -1 -x,-y,-z
      -2 +x,-y+1/2,+z

# ATOMIC COORDINATES AND DISPLACEMENT PARAMETERS


loop_
      _atom_site_type_symbol
      _atom_site_label
      _atom_site_fract_x
      _atom_site_fract_y
      _atom_site_fract_z
      _atom_site_occupancy
      _atom_site_thermal_displace_type
      _atom_site_U_iso_or_equiv
      _atom_site_symmetry_multiplicity
Al
Al1_2e  0.377   0.75     0.126   1.0        Uiso    0.025         2
Al
Al2_2e  0.868   0.25     0.498   1.0        Uiso    0.025         2
Al
Al3_2e  0.875   0.75     0.125   1.0        Uiso    0.025         2
Al
Al4_2e  0.615   0.75     0.745   1.0        Uiso    0.025         2
Al
Al5_4f  0.367   0.075    0.612   1.0        Uiso    0.025         4
Al
Al6_4f  0.116   0.579    0.862   1.0        Uiso    0.025         4
O-
O1_2e   0.881   0.75     0.874   1.0        Uiso    0.025         2
O-
O2_2e   0.614   0.25     0.64    1.0        Uiso    0.025         2
O-
```

```
O3_2e   0.364   0.75     0.889   1.0        Uiso   0.025         2
O-
O4_2e   0.132   0.25     0.627   1.0        Uiso   0.025         2
O-
O5_4f   0.889   0.406    0.899   1.0        Uiso   0.025         4
O-
O6_4f   0.605   0.917    0.614   1.0        Uiso   0.025         4
O-
O7_4f   0.357   0.406    0.853   1.0        Uiso   0.025         4
O-
O8_4f   0.138   0.916    0.637   1.0        Uiso   0.025         4

loop_ _atom_type_symbol
      _atom_type_number_in_cell
                   O- 24.0
                   Al 16.0

# If you change Z, be sure to change all 3 of the following
_chemical_formula_sum                  "Al2 O3"
_chemical_formula_weight               101.96
_cell_formula_units_Z                  8


#--eof--eof--eof--eof--eof--eof--eof--eof--eof--eof--eof--eof--eof--eof-
-eof--#
```

REFERENCES

(1) Ngouana-Wakou, B.F.; Cornette, P.; Corral Valero, M.; Costa, D.; Raybaud, P. An atomistic description of the γ-alumina/water interface revealed by ab initio molecular dynamics. *J. Phys. Chem. C* **2017**, *121*, 10351-10363.

(2) Drecun, O.; Striolo, A.; Bernardini, C. Structural and dynamic properties of some aqueous salt solutions. *Phys. Chem. Chem. Phys*., **2021**, *23*, 15224-15235.
